# A seamless and iterative DNA assembly method named PS-Brick and its assisted metabolic engineering for threonine and 1-propanol production

**DOI:** 10.1186/s13068-019-1520-x

**Published:** 2019-07-15

**Authors:** Shuwen Liu, Haihan Xiao, Fangfang Zhang, Zheng Lu, Yun Zhang, Aihua Deng, Zhongcai Li, Cui Yang, Tingyi Wen

**Affiliations:** 1 CAS Key Laboratory of Pathogenic Microbiology and Immunology, Institute of Microbiology, Chinese Academy of Sciences, Beijing, 100101 China; 2 University of Chinese Academy of Sciences, Beijing, 100049 China; 3 Institute of Physical Science and Information Technology, Anhui University, Hefei, 230039 China; 4 Savaid Medical School, University of Chinese Academy of Sciences, Beijing, 100049 China

**Keywords:** DNA assembly, Metabolic engineering, Design-build-test-learn cycle, Threonine, 1-Propanol, *Escherichia coli*

## Abstract

**Background:**

DNA assembly is an essential technique enabling metabolic engineering and synthetic biology. Combining novel DNA assembly technologies with rational metabolic engineering can facilitate the construction of microbial cell factories. Amino acids and derived biochemicals are important products in industrial biotechnology with wide application and huge markets. DNA assembly scenarios encountered in metabolic engineering for the construction of amino acid and related compound producers, such as design-build-test-learn cycles, construction of precise genetic circuits and repetitive DNA molecules, usually require for iterative, scarless and repetitive sequence assembly methods, respectively.

**Results:**

Restriction endonuclease (RE)-assisted strategies constitute one of the major categories of DNA assembly. Here, we developed a Type IIP and IIS RE-assisted method named PS-Brick that comprehensively takes advantage of the properties of PCR fragments and REs for iterative, seamless and repetitive sequence assembly. One round of PS-Brick reaction using purified plasmids and PCR fragments was accomplished within several hours, and transformation of the resultant reaction product from this PS-Brick assembly reaction exhibited high efficiency (10^4^–10^5^ CFUs/µg DNA) and high accuracy (~ 90%). An application of metabolic engineering to threonine production, including the release of feedback regulation, elimination of metabolic bottlenecks, intensification of threonine export and inactivation of threonine catabolism, was stepwise resolved in *E. coli* by rounds of “design-build-test-learn” cycles through the iterative PS-Brick paradigm, and 45.71 g/L threonine was obtained through fed-batch fermentation. In addition to the value of the iterative character of PS-Brick for sequential strain engineering, seamless cloning enabled precise in-frame fusion for codon saturation mutagenesis and bicistronic design, and the repetitive sequence cloning ability of PS-Brick enabled construction of tandem CRISPR sgRNA arrays for genome editing. Moreover, the heterologous pathway deriving 1-propanol pathway from threonine, composed of *Lactococcus lactis kivD* and *Saccharomyces cerevisiae ADH2*, was assembled by one cycle of PS-Brick, resulting in 1.35 g/L 1-propanol in fed-batch fermentation.

**Conclusions:**

To the best of our knowledge, the PS-Brick framework is the first RE-assisted DNA assembly method using the strengths of both Type IIP and IIS REs. In this study, PS-Brick was demonstrated to be an efficient DNA assembly method for pathway construction and genome editing and was successfully applied in design-build-test-learn (DBTL) cycles of metabolic engineering for the production of threonine and threonine-derived 1-propanol. The PS-Brick presents a valuable addition to the current toolbox of synthetic biology and metabolic engineering.

**Electronic supplementary material:**

The online version of this article (10.1186/s13068-019-1520-x) contains supplementary material, which is available to authorized users.

## Background

Current challenges of metabolic engineering focus on developing cell factories for specific metabolites that can be produced with titres, yields and productivities as high as possible for industrial application [1–3]. Developing new strains that meet the economic requirements for industrial scale production typically requires 6–8 years and over $50 million [4]. The time- and cost-intensive processes of strain development are in part due to the limitations of our knowledge of genetics, physiology and metabolism. It is necessary to perform many rounds of proof-of-concept studies that first implement specific metabolic designs and then generate new knowledge for improved design. The iterative rounds of strain construction and subsequent phenotypic characterization are also called DBTL cycles [4–6]. In each DBTL cycle of metabolic engineering, enzyme screening, chimeric pathway reconstruction, fine-tuning optimization or metabolic bottleneck elimination is performed one by one, and the titre, yield or productivity of the desired metabolites for engineered strains is stepwise improved to high levels.

Amino acids and amino acid-derived biochemicals are widely used in foods, pharmaceuticals, animal feeds, cosmetics, biofuels and materials. With a constantly increasing market of millions of tons and billions of USD per year, amino acid manufacturing is one of the major pillars of industrial biotechnology [7–9]. Amino acids are important building blocks with complex network regulation. Recently, DBTL cycles of rational metabolic engineering have been successfully conducted to construct producers of amino acids and related compounds [10, 11].

The iterative DBTL cycles of metabolic engineering require enabling technologies for iterative DNA assembly, which introduce additional bio-parts into already assembled constructs [12, 13]. DNA assembly methods can be generally classified into two broad categories: RE-based and homology-directed strategies [13–16], the former being more frequently used for iterative DNA assembly [17, 18]. The BioBrick™ standard of RE-based methods is the first DNA assembly strategy that sequentially integrates small basic parts into a large DNA construct [19, 20]. The basic parts are flanked by Type IIP *Eco*RI and *Xba*I restriction sites in the upstream end and by *Spe*I and *Pst*I restriction sites in the downstream end, where *Xba*I and *Spe*I are isocaudomers with two compatible sticky ends. The ligation of the digested parts generates scar sequences that are different from the sequences of both original sites and thus cannot be cut in subsequent digestions with either *Xba*I or *Spe*I. The assembled construct is flanked by the same active cloning sites as the two parent parts, from which the next round of insertion can be repeated. The reusability and simplicity of BioBricks make them the standard DNA assembly framework for the iGEM (international Genetic Engineered Machine) competition. The assembly of genetic parts conforming to BioBrick standards has become a key part of bioengineering [21–23]. Similar to the BioBrick standard, various Type IIP RE-based strategies have been developed, such as BglBrick [24], ePathBrick [22], iBrick [25], C-Brick [17], *Bacillus* BioBrick Box [26], QGA [14], CCTL [27] and YaliBrick [28].

Although the Type IIP RE-based schemes are easy and straightforward to be performed for iterative DNA assembly, the recognition site remains a scar between the joined fragments and is not considered for seamless cloning. The original BioBrick™ design generates an 8-nucleotide scar between parts joined together, which hampers its application for protein fusions. The modified BglBrick standard [24] uses *Bgl*II and *Bam*HI as the isocaudomers instead of *Xba*I and *Spe*I, and thus generates a 6-nucleotide scar sequence (GGATCT) encoding glycine-serine that is suitable for the in-frame fusion of coding sequences. The currently developed assembly schemes derived from BioBrick also leave behind 6–21 bp scars [1, 14, 25, 27–29].

Compared to assembly schemes using Type IIP REs that recognize and cut within a palindromic sequence, Golden Gate assembly [30] utilizes Type IIS enzymes that cut outside of recognition site in a variable sequence that can be customized as overlapping regions for fusions of multiple parts in a predefined order. Despite its multi-part advantage, Golden Gate is limited in reusability. MoClo and Golden Braid variants brought breakthroughs to Golden Gate assembly that enable full reusability of composite parts [12, 31, 32]. Golden Braid frameworks adopt a double loop topology of multi-vector levels to achieve multipartite expansion. On the other hand, the MoClo uses more levels of topology and a complex workflow. To make the resulting parts fully reusable, an indefinite number of additional destination plasmids for subsequent hierarchy levels would be required. The MoClo toolkit was then adapted with modifications to the vectors and schemes for *Escherichia coli* [33], yeast [34], plants [35] and mammalian cells [36]. Taken together, both MoClo and Golden Braid frameworks enable an iterative workflow; however, it requires elaborate plasmid libraries and/or sacrifices multipart assembly [37].

The interstitial scar sequences between joined DNA fragments can be problematic for maintaining DNA integrity and for mRNA folding, increasing the difficulty for sequence design [38, 39]. For precise DNA assembly into genes, circuits, metabolic pathways and even metabolic modules, the consecutive combinations of genetic context-dependent elements (such as enhancers, promoters, RBSs, spacers, genes and terminators) require the development of scar-free ‘seamless’ assembly techniques [6, 15, 40–42].

Here, we developed a Type IIP and IIS restriction endonuclease-assisted BioBrick (PS-Brick) for both iterative and seamless assembly. With the use of the PS-Brick method, we showcase here the successful achievement of iterative DBTL cycles of metabolic engineering for threonine and 1-propanol production, including the release of feedback regulation of the threonine operon, elimination of a metabolic bottleneck, intensification of threonine export, inactivation of threonine catabolism and construction of a 1-propanol pathway. In addition to the iterative character of PS-Brick for sequential strain engineering, the seamless property enabled precise in-frame fusion for codon saturation mutagenesis and bicistronic design. Moreover, the repetitive sequence cloning of PS-Brick enabled the construction of tandem CRISPR sgRNA arrays with the same promoter and terminator.

## Results

### Design and proof-of-concept of PS-Brick assembly scheme

#### The design principles of PS-Brick

**Fig. 1:**
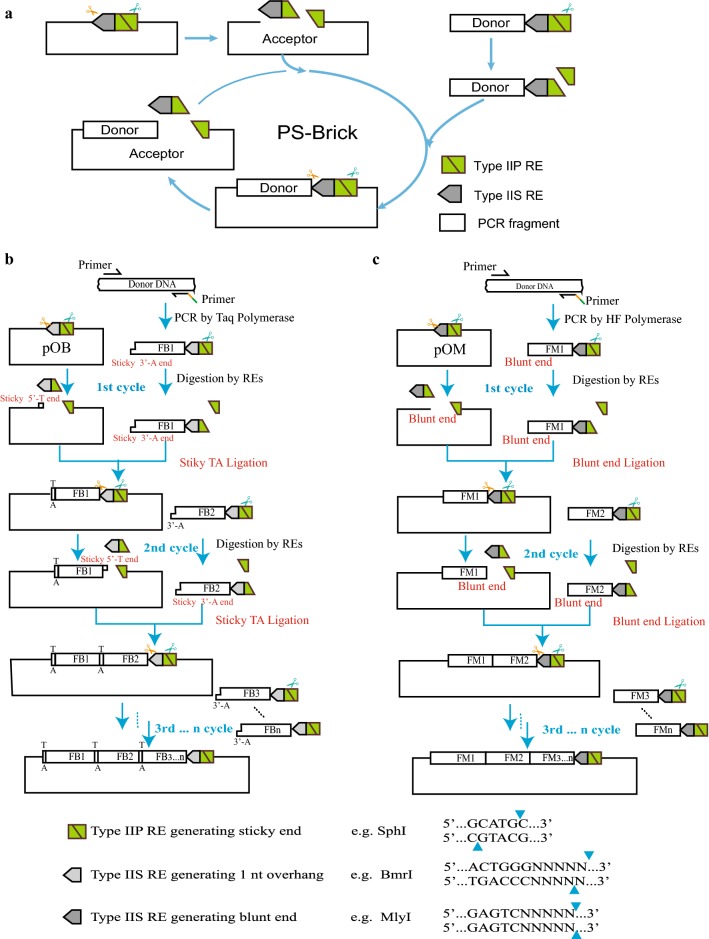
The PS-Brick design and workflow. **a** The overall cycle of the PS-Brick assembly method. **b** Strategy of *Bmr*I based TA cloning. The original PS-Brick vectors pOB containing the entrance sites of adjacent *Sph*I/*Bmr*I were double digested using the corresponding RE pair. The recognition sites of *Bmr*I and half of *Sph*I recognition site were detached, leaving behind one 1-nt cohesive end generated by *Bmr*I and the 4-nt cohesive end generated by *Sph*I. The PCR products amplified by Ex-Taq DNA Polymerase and monoterminally flanked by *Sph*I/*Bmr*I were digested by only *Sph*I, and then linked to the 4-nt complementary cohesive end of the digested vector backbones. Meanwhile, the other end of the digested vector backbones with 1-bp cohesive end was linked with non-cut end of the PCR products through TA cloning. **c** Strategy of *Mly*I based blunt end ligation. The original PS-Brick vectors pOM containing entrance site of adjacent *Sph*I/*Mly*I were double digested using the corresponding RE pair. The recognition site of *Mly*I and half of *Sph*I recognition site were detached, leaving behind a blunt end generated by *Mly*I and a 4-nt cohesive end generated by *Sph*I. The PCR products amplified by Kappa high-fidelity polymerase monoterminally and flanked by *Sph*I/*Mly*I were digested by only *Sph*I, and then linked to the 4-nt complementary cohesive end of the digested vector backbones. Meanwhile, the other end of the digested vector backbones with blunt end was linked with non-cut end of the PCR products through blunt end ligation. The newly assembled vectors once again contains the same entrance sites of adjacent *Sph*I/*Bmr*I or *Sph*I/*Mly*I and could therefore be used for next round of parts incorporation. FB and FM denote the donor PCR fragments for PS-Brick assembly with entrance sites *Sph*I/*Bmr*I and *Sph*I/*Mly*I, respectively

Compared to the original BioBrick standard (using only type IIP REs) and Golden Gate assembly (using only type IIS REs), PS-Brick applies both of them in the assembly reaction (Fig. 1a). The available digested ends created by Type IIS REs include overhangs of up to 4 bp, single base pair overhangs and blunt ends. The PS-Brick scheme adopts Type IIS endonucleases that can generate single base overhangs or blunt ends. Three commercially available Type IIS REs with a single base digestion overhangs (*Bmr*I, *Bci*VI and *Hph*I) have been tested to show sufficient cleavage efficiencies [43]. *Mly*I is the unique Type IIS RE with one recognition site and a blunt digestion product. Considering the frequency and location of these restriction sites in the pUC19 base vector, *Bmr*I (generating 1 nt cohesive end) and *Mly*I (generating blunt end) are used as Type IIS REs for the proof-of-concept of the PS-Brick assembly strategy (Fig. 1bc).

**Fig. 2:**
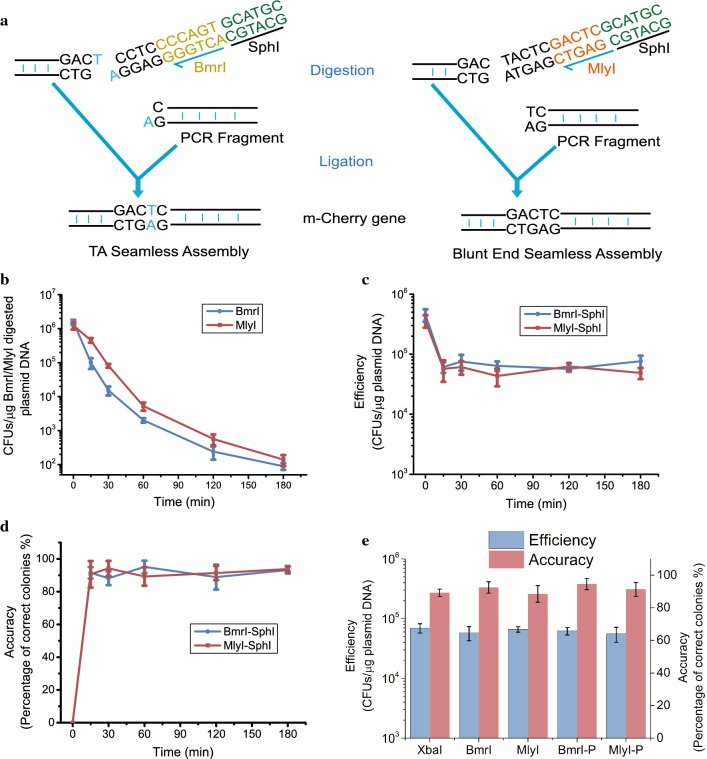
Optimization of the PS-Brick reaction conditions. **a** seamless fusion of mCherry gene through TA cloning or blunt-end ligation based PS-Brick assembly. **b** The transform efficiency of *Bmr*I digested pOB vector and *Mly*I digested pOM vector. **c, d** The second cutting efficiency of *Sph*I was investigated through the assembly efficiency and correct ligation rate. The vector backbones of the *Bmr*I cleaved pOB and *Mly*I cleaved pOM were digested with *Sph*I from 15 min to 180 min, and then linked to PCR products digested by *Sph*I for 180 min. The CFUs after transformation were recorded as assembly efficiency, and the percentage of the correct colonies by DNA sequencing among the total colonies was calculated as assembly accuracy. **e** Comparison of the efficiency and accuracy between PS-Brick and the traditional Type IIP RE-based clone method with XbaI and SphI. The effect 5′-phosphorylation of the non-cut PCR end on the efficiency and accuracy of PS-Brick. Data shown are mean values from three biological replicates, and the standard deviations are presented

One *Bmr*I site and three *Mly*I sites located in the pUC19 vector backbones were removed through overlap extension PCR [44]. One entrance site of adjacent *Sph*I/*Bmr*I or *Sph*I/*Mly*I at the end of truncated *mCherry* was introduced into the mutated pUC19 vector, generating the original PS-Brick vectors pOB and pOM, respectively (see “Methods” section). The truncated site of the *mCherry* gene was located in the unique inverted *Mly*I recognition sequence “GACTC” (Figs. 1 and 2a), and the introduced PCR parts were free of internal *Sph*I, *Bmr*I and *Mly*I sites. The original PS-Brick vectors were double digested using the corresponding REs pair, respectively. The recognition site of the Type IIS REs *Bmr*I or *Mly*I and half of *Sph*I recognition site were detached from the original vector backbones, leaving behind one 1 nt cohesive end generated by *Bmr*I or one blunt end generated by *Mly*I and the other cohesive end (4 nt), which is generated by *Sph*I (Fig. 1b, c). The genetic parts to be joined were defined as donor fragments and were supplied in the form of PCR products. Compared to certain high-fidelity DNA polymerases generating blunt end PCR products, Taq polymerase can add a single non template-directed deoxyadenosine (dA) residue to the 3′ end of duplex PCR products [45]. The dA overhang of amplified DNA fragments can be directly ligated into a single 3′ deoxythymidine (dT) overhang at the end of *Bmr*I-digested pOB (Fig. 1b). The blunt end of donor PCR fragments amplified by high-fidelity polymerase can be ligated to the blunt end of *Mly*I digested vector pOM (Fig. 1c).

Compared to the previous BioBrick system and its derivatives that use donor parts prefixed and suffixed with restriction enzyme sites at both ends, donor PCR fragments in PS-Brick are only single-end flanked by adjacent Type IIP and Type IIS restriction sites (Figs. 1and 2a). The required extensions of adjacent *Sph*I/*Bmr*I, or adjacent *Sph*I/*Mly*I, were designed in primer extensions. The PCR products flanked by the adjacent RE pairs (*Sph*I/*Bmr*I or *Sph*I/*Mly*I) on one end were digested by only *Sph*I, and then linked to the 4-nt complementary cohesive end of the *Sph*I/*Bmr*I double-digested pOB or *Sph*I/*Mly*I digested pOM vector backbones. Meanwhile, the other end of the digested vector backbones with a 1 nt cohesive end or a blunt end is linked with an uncut end of the PCR products through TA cloning or blunt end ligations, respectively. After ligation, the joint between the Type IIS endonuclease (*Bmr*I or *Mly*I) digested end of the vector and the PCR product end without restriction enzyme sites contains no additional scar sequences (Figs. 1b, c and 2a); the other joint, between the vector end and PCR product end that had been both digested by *Sph*I, once again contains the same adjacent *Sph*I and *Bmr*I sites, or adjacent *Sph*I and *Mly*I sites and could, therefore, be used for next round of part incorporation (Fig. 1). Taken together, these results indicate that a seamless and iterative assembly scheme was achieved through the PS-Brick strategy.

Compared to the previous restriction-ligation methods, the novel designs of PS-Brick are as follows: (i) Two types of REs (Type IIP and IIS) are used in PS-Brick, whereas only one Type of REs is applied in BioBrick-like standards or Golden Gate-like assembly, respectively. (ii) The BioBrick standard (*Spe*I, *Pst*I, *Xba*I and *Eco*RI) and methods similar to it, such as BglBrick (*Eco*RI, *Bgl*II, *Bam*HI, and *Xho*I) [24], ePathBrick (*Spe*I, *Xba*I, *Nhe*I, and *Avr*II) [22] and YaliBrick (*Spe*I, *Sal*I, *Xba*I, and *Avr*II) [28] use four defined Type IIP REs in each method (Table 1), whereas PS-Brick can use all the Type IIP REs cleaving to sticky end, which provides broad options. (iii) The PS-Brick standard adopts Type IIS endonucleases generating single base overhangs or blunt ends to match the PCR products with an unpaired dA extension or a blunt end, respectively, while the most frequently used Type IIS REs for DNA assembly have long cohesive ends (usually 4 nt) [12, 30, 32, 46]. (iv) The donor PCR fragments in PS-Brick are only flanked on one end by restriction sites, allowing for simultaneous utilization of the properties of PCR and REs, whereas the donor PCR fragments are fixed with restriction enzyme sites at both ends in BioBrick and Golden Gate-like restriction-ligation standards. (v) The seamless character of PS-Brick is achieved via blunt end cloning or tailorable TA cloning combining the properties of PCR and Type IIS REs. The blunt end or one-base cohesive end of the insert DNA fragments can be generated by PCR with high-fidelity polymerase or Taq polymerase, respectively, while the complementary ends of the linear receptor vector can be generated by Type IIS REs with a blunt or one-base overhang digestion site, respectively. And (vi) the iterative assembly character of PS-Brick is achieved via one uncleavable joint of blunt end cloning or TA cloning along with a cleavable junction between the Type IIP digested end of receptor plasmid and donor PCR fragments. The recognition site of the Type IIS REs and portions of the Type IIP RE site nucleotides detached from the original vector backbones after double REs digestion. The same adjacent Type IIP and IIS restriction pair site is incorporated into the primer design as the unique entrance used for the next round of assembly.

**Table 1:** The collections of endonuclease-assisted DNA assembly methods

Method	REs used in the method^b^	RE Sub-type	Scar sequence	Iterative Assembly
BioBrick [19]	*Spe*I ∩ *Pst*I ∩ *Xba*I ∩ *Eco*RI	IIP	8 bp	Yes
BglBrick [24]	*Eco*RI ∩ *Bgl*II ∩ *Bam*HI ∩ *Xho*I	IIP	6 bp	Yes
ePathBrick [22]	*Spe*I ∩ *Xba*I ∩ *Nhe*I ∩ *Avr*II	IIP	6 bp	Yes
YaliBrick [28]	*Spe*I ∩ *Sal*I ∩ *Xba*I ∩ *Avr*II	IIP	6 bp	Yes
Quick Gene Assembly [14]	*Spe*I ∩ *Eco*RI ∩ *Xba*I	IIP	6 bp	Yes
iBrick [25]	I-*Sce*I ∩ PI-*Psp*I	Homing endonucleases	21 bp	Yes
C-Brick [17]	FnCpf1 endonuclease	CRISPR–Cas nucleases	6 bp	Yes
CCTL [27]	FnCpf1 endonuclease	CRISPR–Cas nucleases	> 8 bp	Yes
PODAC [13]	*Bsa*I	IIS	8 bp	Yes
Golden Gate [30]	*Bsa*I	IIS	4 bp	No
GoldenBraid [12]	*Bsa*I ∩ *Bsm*BI	IIS	4 bp	Yes^c^
MoClo [32]	*Bsa*I ∩ *Bpi*I	IIS	4 bp	Yes^c^
Start-Stop Assembly [42]	*Sap*I ∩ *Bsa*I ∩ *Bbs*I	IIS	Scarless	Yes
PS-Brick^a^	(One of hundreds of IIP REs generating overhangs of two or more bases) ∩ (*Bmr*I ∪ *Bci*VI ∪ *Hph*I ∪ *Mly*I)	IIP and IIS	Scarless	Yes

^a^This study

^b^∩ Denotes all of the REs before and after this logical AND symbol are simultaneously used, and ∪ denotes only one among the REs before and after this logical OR symbol is used. For example, “*Spe*I ∩ *Pst*I ∩ *Xba*I ∩ *Eco*RI” indicates that the four given REs are simultaneously used in BioBrick standard, and all of the four RE sites are required to be eliminate from the insertion parts. “*Bmr*I ∪ *Bci*VI ∪ *Hph*I ∪ *Mly*I” indicates that any one of the four REs can be adopted in the PS-Brick, providing broad available options

^c^Elaborate plasmid libraries and complex protocols are required for iterative workflows of Golden Braid and MoClo systems

#### Optimization of the PS-Brick

The cutting efficiencies of the Type IIS REs *Bmr*I and *Mly*I presented in vectors pOB and pOM were tested through gel electrophoresis with a cutting time ranging from 15 to 180 min. As shown in Fig. 2bc, almost all of the vectors were digested by *Bmr*I and *Mly*I at 15 min as recommended by the Time-Saver RE protocol. Due to very small amounts of uncut plasmid having a significant impact on transformation, the cutting efficiency of *Bmr*I and *Mly*I was further differentiated by a transformation test. The *Bmr*I-digested pOB and *Mly*I digested pOM were transformed into *E. coli* DH5α competent cells (transformation efficiency with circular pUC19 plasmids: (1.17 ± 0.19) × 10^6^ CFU/µg DNA). As expected, the transformation efficiency gradually reduced (Fig. 2b), indicating that the unobservable uncut plasmids were digested over time. Therefore, the bands of *Bmr*I cleaved pOB and *Mly*I cleaved pOM at 30 min in the gel (Additional file 1: Fig. S1) were recovered for the second *Sph*I digestion. The cutting efficiency of *Sph*I was investigated using the assembly efficiency and correct ligation rate. The vector backbones of the *Bmr*I-cleaved pOB and *Mly*I cleaved pOM were digested with *Sph*I for 15 min to 180 min, and then inactivated at 65 °C, recovered and linked to PCR products digested by *Sph*I for 180 min. The ligation mix was chemically transformed into competent cells. The total colony-forming units (CFUs) after transformation was recorded as assembly efficiency, and the percentage of the correct colonies by DNA sequencing among the total colonies was calculated as the assembly accuracy (also called fidelity or the positive rate) [47, 48]. In both cases, *Sph*I digestion from 15 to 180 min resulted in similar assembly efficiency, approximately 4 ~ 8×10^4^ colony-forming units CFU/µg DNA (Fig. 2c). The accuracy of PS-Brick assembly was measured by DNA sequencing of 20 clones and ranged from 88.2 to 95.1% with varying cutting times for both TA PS-Brick with pOB and blunt end ligations PS-Brick with pOM (Fig. 2d). The seamless joint between the vector and insert was confirmed by DNA sequencing and did not show any mutations. The cutting time course analysis of electrophoresis, assembly efficiency and accuracy indicated that 15 min was enough for both Type IIS REs and the following Type IIP REs digestion in PS-Brick. Considering the two DNA recoveries for 30 min each, RE inactivation for 20 min, DNA ligation for 30 min, *E. coli* transformation for 30 min and incubation for 40 min, the PS-Brick workflow can be performed within half of a working day (Additional file 1: Fig. S2). Each round of PS-Brick (from amplification of DNA fragments to clone verification by PCR) can be finished in two working days (Additional file 1: Fig. S2).

To compare this method with the traditional Type IIP RE-based cloning methods, the same *mCherry* fragment and the *Sph*I and *Xba*I sites present in the MCS sequence of the pUC19 vector were used in the PS-Brick assembly standard. The assembly efficiency and accuracy of traditional clone method with *Sph*I and *Xba*I were similar to those of the PS-Brick (Fig. 2e). The three methods used the same *Sph*I site at one end of both PCR products and linearized vector, so the other end with 4 nt overhangs generated by *Xba*I, 1 nt overhangs generated by *Bmr*I and blunt ends generated by *Mly*I resulted in similar ligation efficiency. However, cohesive end ligation and TA cloning were expected to be more efficient than blunt end ligation [45]. The difference may arise from the different enzymes used for vector preparation [43]. Preferably, enzymes with extensive usage history, high efficiency and non-methylation are suggested to be adopted in the PS-Brick standard.

A single TA base pair can be easily found in most DNA. Seamless TA fusions require the design of the cutting sites of *Bmr*I to contain dT/dA bases in the insert/vector transition (Fig. 1b, c), whereas the blunt end/*Mly*I-based PS-BioBrick strategy allows complete seamless assembly. The blunt end/*Mly*I-based ligations require no A-tailing step of PCR products but reach similar ligation efficiencies as TA cloning and allow complete sequence-independent fusions. In this respect, blunt end ligation-based PS-Brick is completely sequence-independent and thus better suited for universal application. In another respect, only one Type IIS Re *Mly*I can be used for blunt end ligation-based PS-Brick, whereas three REs (*Bmr*I, *Bci*VI, *Hph*I) can be used for TA clone-based PS-Brick, suggesting that TA clone-based PS-Brick has more options for RE selection. Taken together, these requirements indicate that the user can apply alternative PS-Brick schemes according to the practical demands of their projects.

The clones that failed to assemble an insert were also confirmed by DNA sequencing and showed the same sequence as the original vectors pOB and pOM, indicating that the linearized vectors after digestion by *Bmr*I and *Sph*I were not thoroughly digested by *Sph*I and then self-ligated. To improve the assembly accuracy, dephosphorylation by alkaline phosphatase is necessary to counteract the self-ligation of the vector; however, it would prolong the durance and complicate the workflow of PS-Brick. The 5′-phosphorylated on the non-cut PCR end as another method can be used for efficient ligation, but the accuracy of PS-Brick was not found to improve when primers for insert amplification were phosphorylated prior to ligation (Fig. 2e). Moreover, the price of a 5′-phosphorylated primer was 5.9–7.9 times higher than that of the non-phosphorylated primer containing the same 23 nucleotides synthesized by Thermo Fisher Inc. Considering that the assembly efficiency and accuracy for PCR insertion without phosphorylation and with dephosphorylation for vector backbones were still sufficient for most projects, the PS-Brick scheme did not include these two steps and made it simpler and more cost effective.

### DBTL cycles for pathway engineering for the production of threonine and its derivate 1-propanol through PS-Brick

**Fig. 3:**
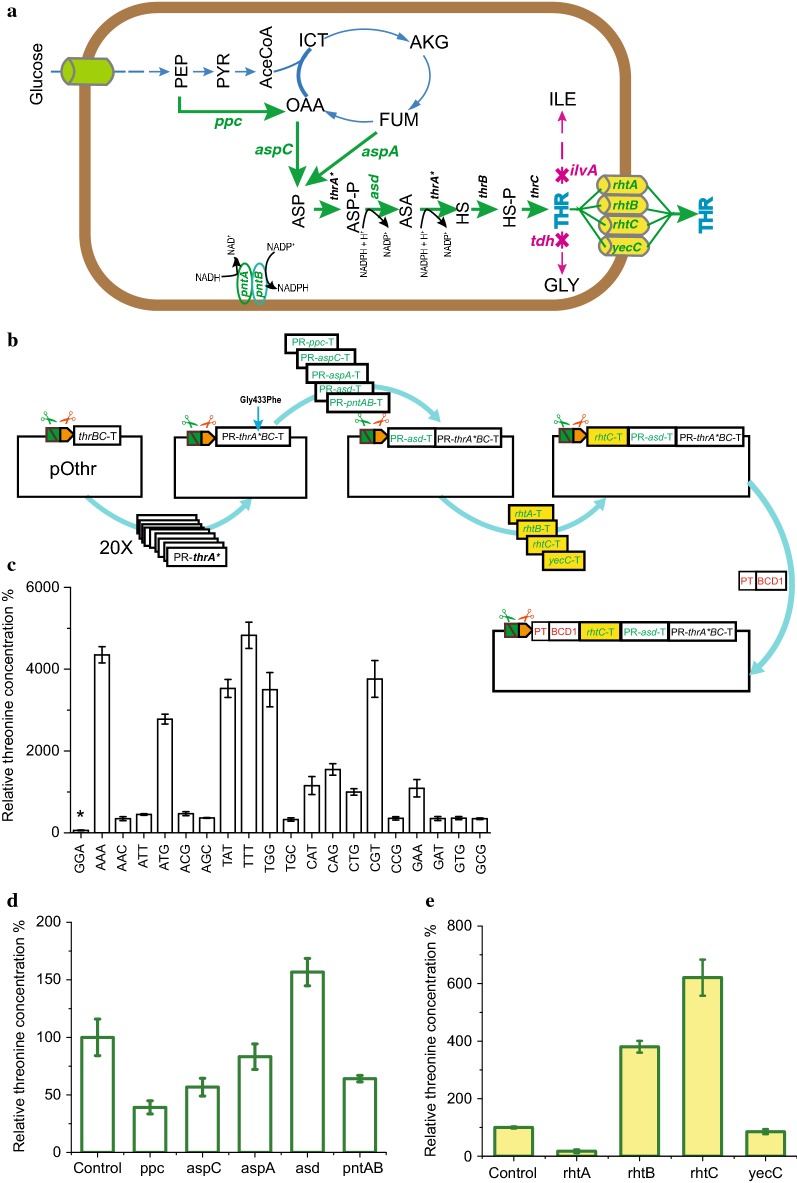
DBTL cycles of threonine pathway engineering through the PS-Brick scheme. **a** The metabolic pathway for threonine synthesis from glucose. **b** Three rounds of DBTL cycle of metabolic engineering were performed step by step through four iterative rounds of PS-Brick assembly from pOthr vector. The most efficient mutant of feedback-resistant ThrA was screened through the first round of PS-Brick. The bottleneck gene for threonine synthesis was identified and overexpressed through the second round of PS-Brick. Four threonine exporters under the control of the same promoter and RBS were prioritized through the third and fourth rounds of PS-Brick. PR indicates the native promoter and RBS, and T indicates the native terminator. **c** The threonine production with 20 ThrA mutants. The assembled plasmids harbouring the thrABC operon containing the saturation mutated *thrA* gene (pACYC184-*thrA*^*433*^*BC*) were transformed into the *E. coli* K12 MG1655 strain, respectively. The threonine titers at the 12 h of shake flask fermentation were measured. *Denotes the wild-type control. **d** Effects of the *ppc*, *aspA*, *aspC*, asd and *pntAB* genes overexpression on the threonine production. The assembled plasmids pACYC184-*thrA*^*433*^* BC-asd/ppc/aspA/aspC/pntAB* were transformed into the *E. coli* K12 MG1655 strain, respectively. The threonine titres at the 12 h of shake flask fermentation were measured. **e** Effects of overexpression of the four exporters on the threonine production. The assembled plasmids pACYC184-*thrA*^*433*^*BC-asd*-P_T_BCD1-* rhtA/rhtB/rhtC/yeaS* were transformed into the *E. coli* K12 MG1655 strain, respectively. The threonine titres at the 12 h of shake flask fermentation were measured. Prioritization of isoenzymes for threonine efflux. Promoter (P_T_) and RBS (BCD1) was inserted in front of start codon ATG of the four exporter gene. The relative concentration was calculated by dividing the measured concentration of threonine produced with the engineered strains by that with control strain in the same batch of flask fermentation. Data shown are mean values from three biological replicates, and the standard deviations are presented

Many optimal solutions of metabolic engineering are resolved in a stepwise manner by repeated DBTL cycles [4, 5]. As a showcase for the PS-Brick method, we constructed cell factories overproducing threonine with iterative DBTL cycles. The general strategy for the development of a genetically defined threonine overproducing strain includes release of feedback regulation on the threonine operon, intensification of biosynthesis pathways, elimination of rate-limiting steps, inactivation of threonine catabolism, modification of product transport and finally reinforcement of the regeneration of coenzymes (Fig. 3a) [10, 11, 49]. The optimal solutions of each procedure of the metabolic engineering strategy were resolved by one DBTL cycle. Four rounds of the DBTL cycle were performed step by step through six cycles of PS-Brick reactions, and the threonine production with engineering strains gradually increased to higher levels.

#### Site-directed mutagenesis for feedback-resistant ThrA

The seamless assembly of PS-Brick was expected to perform codon saturation mutagenesis (mutagenesis that causes a change from a wild-type amino acid to all other amino acids) by varying the sequence in the primer of the PCR insert. To demonstrate the feasibility, we edited the bifunctional enzyme aspartokinase I–homoserine dehydrogenase I (AC1–HDH1), encoded by the *thrA* gene, to relieve the feedback inhibition by threonine (Fig. 3a). Mutations in ThrA to release feedback inhibition are the most important modification for threonine production in *E. coli* [11, 49]. A feedback-resistant ThrA variant (Gly433Arg) derived from the threonine hyper-producer ATCC 21277 [50] has been widely used for the production of threonine and its derivatives [51–53]. In addition to the wild-type Gly and the mutated Arg at the 433th residue of ThrA, all other 18 amino acids, encoding by the codons with high usage frequency in *E. coli* K12 MG1655, were designed as the alternatives in this study (Fig. 3b).

For PS-Brick assembly, the Type IIP RE *Hin*dIII and the Type IIS RE *Mly*I were used as the iterative RE pair. There are four *Mly*I sites located in the pACYC184; thus, this vector cannot be directly used for PS-Brick assembly. The entrance site of *Hin*dIII/*Mly*I was introduced into the medium-copy-number plasmid pACYC184 to generate original vector pOthr for PS-Brick assembly. Three *Mly*I sites located in the pACYC184 vector backbones were mutated through overlap extension PCR with primers AC3211-F/AC727-R and AC727-F/AC1143-R. Another *Mly*I site located in the MCS sequence of the pACYC184 vector was removed by *Hin*dIII and *Nru*I digestion. The remaining pACYC184 vector backbone without the *Mly*I site was linked with PCR products of the truncated *thrABC* operon (a part of thrA and the entire thrBC with the native terminator) by primers TAB-F/TAB-R, TBC-F/TBC-R and TC-F/TC-R. The *Nru*I site was designed in a primer outside of the terminator of the *thrC* gene, and the *Hin*dIII site was designed outside of the truncated *thrA* and internally adjacent to the *Mly*I site (Fig. 3b). After ligation and sequencing verification, the newly obtained vector pOthr, containing adjacent *Hin*dIII/*Mly*I entrance sites, was used for subsequent in-frame fusion of ThrA (Fig. 3b).

The truncated site of the *thrABC* operon is located in the target *thrA*^Gly433^ codon sequence. The sites of codon saturation mutagenesis of the 433th residue of ThrA were designed in 20 inverse primers of the PCR donors, and the adjacent *Hin*dIII/*Mly*I site was designed in the extension of one forward primer (Fig. 3b). The 20 PCR donor fragments, including promotor and different mutated *thrA*^Gly433^, were incorporated into the acceptor vector pOthr. Thus, 20 mutants (including promoter, RBS, *thrA*^Gly433^BC and terminator) were generated by one cycle of PS-Brick assembly. Meanwhile, the joint between vector end and the PCR product end that were both digested by *Hin*dIII once again contains the same adjacent *Hin*dIII/*Mly*I sites and could, therefore, be used for next round of parts incorporation (Fig. 3b).

After verification by DNA sequencing, the correct plasmids harbouring the *thrABC* operon containing the saturation mutated *thrA* gene (pACYC184-*thrA*^*433*^*BC*) were transformed into the *E. coli* K12 MG1655 strain. The overexpression of 20 ThrA variants resulted in a significant difference in threonine production after 12 h of shake flask fermentation (Fig. 3c). Remarkably, the *thrA*^Gly433Phe^ mutant resulted in the highest concentration of threonine, 48-fold higher than that of the wild-type control. The efficient desensitized ThrA^Gly433^ variant identified through saturation mutagenesis can be widely used for the development of a producer of threonine and its derivatives.

#### Identification of the metabolic bottleneck for threonine synthesis

It is well known that overexpression of deregulated *thrABC* is usually the most productive strategy for metabolic engineering of threonine [49, 54, 55]. However, the metabolic bottleneck after overexpression of *thrABC* for efficient threonine synthesis was unclear. The production of threonine could be increased through up-modulation of the following genes directly related to the threonine-synthesis pathway (Fig. 3a): the *ppc* gene encoding phosphoenolpyruvate carboxylase, the *pntAB* operon responsible for the regeneration of reduced NADPH in *E. coli*, the *aspC* gene encoding aspartate aminotransferase for transferring the amino group, the *aspA* gene encoding aspartase responsible for the return of carbon from the threonine biosynthetic branch to the tricarboxylic acid cycle and the *asd* gene encoding aspartyl semialdehyde dehydrogenase. However, the contributions of these genes to threonine accumulation were not simultaneously compared under the same experimental conditions. Here, *ppc*, *aspA*, *aspC*, *asd* and *pntAB* with their native promoters, RBSs and terminators were amplified as donor PCR fragments and then assembled in parallel into the last round assembled vector pACYC184-*thrA*^*433*^*BC* using PS-Brick to investigate the potential rate-limiting step for threonine accumulation (Fig. 3b).

The metabolic bottleneck after overexpression of *thrABC* was identified through the second round of PS-Brick assembly. After sequencing verification, the correct plasmids pACYC184-*thrA*^*433*^*BC*-*ppc/aspA/aspC/asd/pntAB* were transformed into the *E. coli* K12 MG1655 strain. Transcriptional analysis of the *ppc*, *aspA*, *aspC*, *asd* and *pntAB* genes by real-time RT-PCR indicated that the transcriptional levels of all these genes were significantly increased (Additional file 1: Fig. S3). Among the proposed five rate-limiting steps, only overexpression of the *asd* gene led to a 56.7% increase in threonine production, compared to that obtained with the control strain (Fig. 3d). By contrast, the high expression of Asd had little effect on threonine accumulation in *Corynebacterium glutamicum* [56]. The *asd* gene encoding aspartyl semialdehyde dehydrogenase was first demonstrated as the metabolic bottleneck after *thrABC* overexpression in this study. This engineering target was unable to be predicted purely though metabolic analysis, mainly due to a significant lack of a priori knowledge about the interactions between the target reaction and the intricate metabolic network with its innumerable components [6, 54, 57]. Therefore, it is often necessary to try several versions of a construct to find the optimal configuration. The iterative nature of PS-Brick enables one to subsequently identify major bottlenecks and gradually optimize strain performance, while simultaneously conserving the positive construct.

However, threonine production with strains overexpressing *ppc*, *aspC*, *aspA* and *pntAB* decreased by 60.8%, 43.3%, 16.7% and 35.9%, respectively, compared to production with the control strain MG1655/pACYC184-*thrA*^*433*^*BC* (Fig. 3d), which is consistent with the previous report that overexpression of the *ppc* gene in a plasmid decreased threonine production [58]. The increased activity of anaplerotic phosphoenolpyruvate carboxylase coupled with the simultaneous activity of gluconeogenic reactions will make up ATP-dissipating futile cycles [59], which was assumed to deteriorate threonine synthesis with its high energetic costs [60]. In the case of *pntAB* overexpression, the pathways of NADPH oxidation can be activated and lead to the formation of futile cycles in *E. coli*, which also consumes energy required for both cell growth and threonine synthesis [49]. The negative effects of overexpression of the four genes suggested that upregulation of the non-rate-limiting reaction step of pathway inversely decreases threonine accumulation.

#### Prioritization of isoenzymes for threonine efflux

As another case of trial and test, PS-Brick was used to prioritize isoenzymes for threonine efflux. Efficient export of threonine is also important to further increase its production. The intracellular concentration of threonine was tenfold higher than that observed in the medium during the growth phase, implying the necessity for accelerating threonine export [61]. Overexpressing the exporter genes not only accelerates threonine secretion, but also reduces its consumption [55, 62]. Three exporter genes, *rhtA*,* rhtB* and* rhtC*, have been overexpressed together in a plasmid for development of a threonine producer [58, 63], but data for comparing the capacity of these transporters in *E. coli* are scarce [64]. In our previous work, we found that the high expression level of membrane transporter would inhibit cell growth (PCT Patent WO2015197014A1). Here, mid-strength transcription and translation initiation elements were used for expression of the membrane exporters. Four threonine exporters under the same standard initiation element (a mid-strength promoter (P_T_) from a constitutive bacteriophage P_L_ promoter library [65] along with a mid-strength bicistronic design (BCD1) RBS [66]) were assembled into the last round PS-Brick plasmids pACYC184-*thrA*^*433*^*BC-asd* through two cycles of PS-Brick reaction, respectively. First, each coding sequence of the *rhtA*, *rhtB*, *rhtC* and yeaS genes with their native terminator was amplified as one donor PCR fragment and inserted into the pACYC184-*thrA*^*433*^*BC-asd* vector through PS-Brick. Second, P_T_ and BCD1 [65, 66] were amplified as donor PCR fragments, and inserted in front of the ATG start codon of the four exporter genes in the last round assembled vectors, generating the corresponding vectors pACYC184-*thrA*^*433*^*BC-asd*-P_T_BCD1-*rhtA/rhtB/rhtC/yeaS *(Fig. 3b). Remarkably, the seamless character of PS-Brick guaranteed precise fusion between the BCD translation initiation element and the downstream coding sequence. The stop codon UAA in a first cistron of BCD1 overlaps the start codon AUG of the downstream coding sequence of the four exporters by 1 base pair, leading to both a stop and start codon via a − 1 frame shift (UAAUG) [66].

After sequencing verification, the correct plasmids containing threonine efflux genes were completed by a fourth cycle of PS-Brick. Transcriptional analysis of the *rhtA*, *rhtB*, *rhtC* and *yeaS* genes by real-time RT-PCR indicated that expression of all the genes was significantly upregulated (Additional file 1: Fig. S4). The result of shake flask culture showed that the overexpression of each of the four exporter genes resulted in a distinct increase in threonine production. Among them, the overexpression of the *rhtC* gene increased threonine production from 0.9 g/L to 3.7 g/L (Fig. 3e), which was consistent with previous results showing that the heterologous expression of *E. coli* exporters could increase threonine production in *C. glutamicum* strains [67]. Thus, the assembled plasmid containing *rhtC* was used for the following threonine production.

#### Construction of a CRISPR array containing sequence repeats

**Fig. 4:**
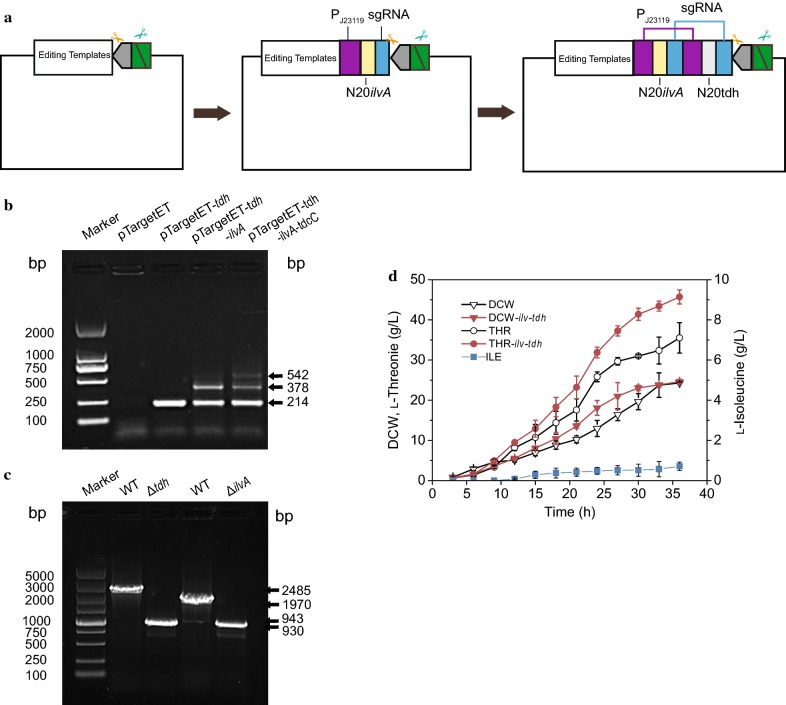
Sequence repeat assembly through the PS-Brick scheme. **a** Construction of CRISPR array containing sequence repeats via TA clone/BciVI based PS-Brick assembly. The N20 sequence flanked with the same promoter pJ23119 and the sgRNA sequence located in the pTargetET vector. The entrance site of *Hind*III/*Bci*VI and editing template was introduced into pTargetF to generate original vector ptargetET for PS-Brick assembly. The N20 sequences of *tdh* and *ilvA* fixed with the same promoter and sgRNA at each end were sequentially inserted into ptargetET at twice PS-Brick reaction. **b** Identification of sgRNA arrays constructed into the pTarget vector. **c** Identification of *tdh* and *ilvA* gene deletion. **d** Fed-batch fermentation of the engineering strain in 7.5 L fermentor. Strain MG1655△*tdh*△*ilvA*/pACYC184-*thrA*^*433*^*BC-asd*-P_T_BCD1*-rhtC* (solid symbol), strain MG1655/pACYC184-*thrA*^*433*^*BC-asd*-P_T_BCD1-*rhtC* The control strain (open symbol), biomass (triangle), threonine (circle) and isoleucine (square). Data shown are mean values from three replicates, and the standard deviations are presented

In the above case studies, the blunt end/*Mly*I based PS-Brick strategy was used to gradually combine the positive effects of the key genes for threonine accumulation. In this case study, a TA clone/*Bci*VI-based PS-Brick strategy was used to create sgRNA arrays to delete the genes responsible for threonine catabolism. In *E. coli*, threonine catabolism is mainly catalysed by threonine dehydrogenase (encoded by *tdh* gene) and threonine deaminase (encoded by *ilvA*). The deletion of the *tdh* [53, 58, 68] and *ilvA* genes [69] is used in creating threonine producers. To this end, we extended the application to a CRISPR–Cas9 genome editing system for gene deletion. This system included two plasmids, pCas (harbouring the *cas9* gene) and pTarget (carrying the sgRNA recognizing the targeted region) [70]. The pTarget vector consisted of a strong constitutive promoter pJ23119 (http://parts.igem.org/Part:BBa J23119), an N20 sequence and an sgRNA scaffold sequence (Fig. 4a). For double gene deletion, two sgRNA arrays with the same promoter and sgRNA scaffolds are required to be constructed into the pTarget vector.

Homology-based assembly methods often fail to construct vectors containing extensive repeated sequences [13, 24, 71]. In this study, the PS-Brick scheme was used to assemble repetitive sgRNA arrays, and the Type IIP RE *Hind*III and the Type IIS RE *Bci*VI were used as the iterative RE pair. The entrance site of *Hind*III/*Bci*VI and the editing template was introduced into pTargetF to generate the original vector ptargetET for PS-Brick assembly. The N20 sequences of tdh and *ilvA* with the same promoter and sgRNA at each end were sequentially inserted into ptargetET through two cycles of the PS-Brick reaction, resulting in ptargetET-*tdh-ilvA* consisting of double sgRNAs (Fig. 4a, b).

Following the genome editing protocol [70], we transformed ptargetET-*tdh-ilvA* into a strain expressing Cas9, and the double mutation strain *E. coli* MG1655△*tdh*△*ilvA* was obtained (Fig. 4c). After three rounds of iterative digestion and ligation, we successfully assembled up to three copies of sgRNA arrays (Additional file 1: Fig. S5), but the triple mutation was not screened. The recombinant plasmid pACYC184-*thrA*^*433*^*BC-asd*-P_T_BCD1-*rhtC*, integrating the positive effects accumulated through four rounds of PS-Brick, was transformed into the *E. coli* MG1655△*tdh*△*ilvA* strain. Batch culture of this engineered strain in a 7.5-L fermenter allowed production of 45.71 g/L threonine, 28.7% higher than the production of control strains retaining the *tdh* and *ilvA* genes (35.52 g/L), and no isoleucine was detected during the fermentation process (Fig. 4d), suggesting that the prevention of threonine consumption is an effective strategy for its accumulation.

#### Construction of the heterologous 1-propanol pathway

**Fig. 5:**
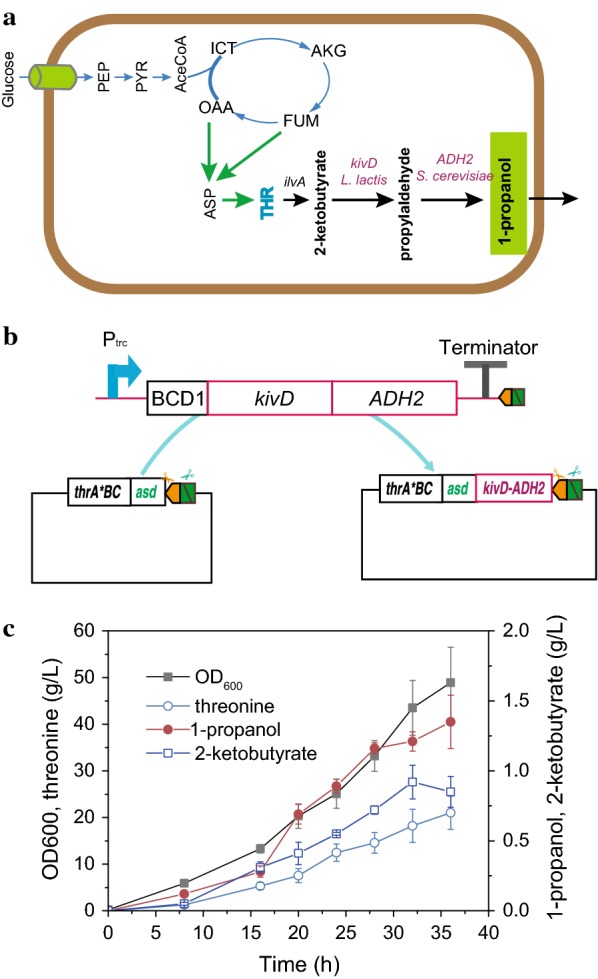
Heterologous 1-propanol pathway engineering. **a** The heterologous 1-propanol pathway containing *Lactococcus lactis kivD* (encoding 2-keto-acid decarboxylase) and *Saccharomyces cerevisiae ADH2* (encoding alcohol dehydrogenase). **b** Heterologous *kivD* and *ADH2* under the P_trc_ and BCD1 control in an artificial operon were assembled in threonine-producing vector p*hrA*^*433*^*BC-asd* through one cycle of PS-Brick reaction. **c** Fed-batch fermentation of the engineering strain MG1655/p*thrA*^*433*^*BC-asd-kivD-ADH2* in 7.5 L fermentor. Data shown are mean values from three replicates, and the standard deviations are presented

1-propanol serves as a promising alternative biofuel and an important solvent and chemical for industrial applications [72, 73]. However, the production of 1-propanol is still dependent on petroleum. In this study, we assembled a heterologous route for 1-propanol production from threonine in *E. coli* [51] (Fig. 5a). *Lactococcus lactis kivD* (encoding 2-keto-acid decarboxylase) and *Saccharomyces cerevisiae ADH2* (encoding alcohol dehydrogenase) under the P_trc_ and BCD1 [66] control in an artificial operon were assembled in threonine-producing vector phrA^433^BC-asd through one cycle of PS-Brick (Fig. 5b). After sequencing verification, the correct plasmid p*thrA*^*433*^*BC-asd-kivD-ADH2* was transformed in* E. coli* MG1655. The expression of *kivD* and *ADH2* genes gave rise to the accumulation of 1.35 g/L 1-propanol in 36 h (Fig. 5c). Meanwhile, the precursor threonine and 2-ketobutyrate accumulated at relatively high concentrations of 21.01 g/L and 0.89 g/L, respectively, which suggests that the activity of heterologous KivD and ADH2 was not high enough. In future work, further effort should be made to obtain 2-keto-acid decarboxylase and alcohol dehydrogenase with high efficiency.

## Discussion

DNA synthesis and assembly are the major enabling technologies that contribute to the foundational innovations of synthetic biology [6, 16, 74]. Here we have developed a novel PS-Brick framework that enables iterative, seamless and sequence-repeat DNA assembly. The core principle of PS-Brick is the comprehensive exploitation of the properties of PCR products and Type IIP and IIS REs. To the best of our knowledge, the PS-Brick framework is the first restriction-ligation assembly method using both Type IIP and IIS REs (Table 1). Type IIP RE-assisted BioBrick standards and variants are limited in scarless assembly, whereas Golden Gate assembly [30] utilizes only Type IIS enzymes and is limited in reusability. Although MoClo and Golden Braid variants brought breakthroughs into the Golden Gate assembly that enabled iterative workflows, they require substantial efforts to prepare elaborate assembly vectors and complex protocols [12, 32], which poses a barrier to their widespread adoption [42]. PS-Brick assembly is an attempt to extend the capabilities of the previously described RE-based strategies to satisfy the requirements of synthetic biology and metabolic engineering.

First, there is a requirement for iterative assembly. Due to the high complexity of the extensive regulation and interactions among the metabolic, gene regulatory and signalling networks, and the limited a priori knowledge for predicting how well a given DNA construct will work once introduced into a cell, it is often necessary to test numerous versions of the construct to find the optimal version [6, 57]. Therefore, it is crucially important to perform sequential DBTL engineering cycles in a proof-of-concept study, which requires a simple and streamlined DNA assembly framework with iterative properties.

Second, there is a requirement for seamless assembly. The interstitial scar sequences between joined DNA fragments can be problematic for maintaining DNA integrity and mRNA folding, which increases the difficulty of sequence design [38, 39]. When a large DNA molecule is assembled stepwise into a backbone plasmid in a random piecewise manner, the scars damage the structure of the original DNA sequence in the final assembled plasmids [75]. The precise combinations of genetic context-dependent elements (such as enhancers, promoters, RBSs, spacers, protein domains and terminators) require the development of scarless assembly processes [6, 15, 40–42].

Third, there is a requirement for the assembly of repetitive sequences. DNA molecules with repetitive sequences, such as the TALEN DNA-binding modules [76], polyketide modules [77], CRISPR array [13] and any DNA sequence that appears more than once in insertions [46]. Assembly of these tandem repeat sequence can be problematic for cloning techniques based on PCR and sequence homology [13, 24, 71]. There are two main reasons for this limitation. On the one hand, misalignment and erroneous annealing of primers makes it difficult to create repetitive sequences using PCR-based methods. However, parts containing approximately the same sequence can be targets for recombination, which can often lead to deleted and/or rearranged DNA components [24]. RE-based assembly is a particularly attractive strategy for the construction of DNA components with identical elements.

As a demonstration for iterative, seamless and repetitive sequence assembly, we applied PS-Brick to design iterative DBTL cycles of metabolic pathway construction and optimization for cell factories overproducing threonine and its derivate 1- propanol. An expression vector was assembled in four rounds of PS-Brick reactions, among which seamless character was highlighted when the in-frame fusion of ThrA for codon saturation mutagenesis and precise BCD assemble between a stop codon and an initiation codon. We have also succeeded in assembling a CRISPR-Cas9 genome editing plasmid containing three sequence repeats of sgRNA arrays in three rounds of reactions. The highest titres of threonine with *C. glutamicum* and *E. coli* engineered from the wild-type strains reached 12.8 g/L and 82.4 g/L, respectively [56, 58]. In this study, 45.71 g/L threonine was obtained by deleting two genes and overexpressing two genes and one operon. The 1-propanol titre was still relatively low than the highest concentration of 10.8 g/L obtained in 120 h with engineered *E. coli* [78]. In future studies, efficient technologies, such as biosensors, chromosomal integration, combinatorial and fine-tuning strategies [9, 10], should be applied to improve the yield of the strains.

In addition to the three key properties of seamless, iterative and repetitive sequence assembly, PS-Brick technology has the following advantages. A first comparative advantage is its efficiency and accuracy: many restriction endonucleases fail to cleave when their recognition sequences are located within a few base pairs of the end of a DNA fragment [79]. Here, the frequently used and high-efficiency Type IIP REs (e.g. *Hind*III and *Sph*I) that generate long sticky ends were used for cutting the donor PCR fragments and linear recipient vector, the end of which contained the corresponding recognition sequences. Furthermore, the infrequent Type IIS REs (*Mly*I, *Bmr*I and *Bci*VI) were used only for digesting the circular plasmid, and the thoroughly linearized plasmid was used for the second digestion with the above high-efficiency Type IIP REs. The intrinsic robustness of the restriction enzymes involved in the PS-Brick reaction might lead to both high efficiency (10^4^–10^5^ CFU/µg DNA) and accuracy (~ 90%). A second advantage is low-cost and speed: long oligonucleotides, phosphorylated or methylated PCR products and dephosphorylated vector backbones are unnecessary in this method, which reduces the cost of the PS-Brick process. The time-saving commercial REs, such as FastDigest REs from New England Biolabs Inc., QuickCut REs from TaKaRa Bio Inc. and Time-Saver REs from Thermo Fisher Inc., could digest the DNA substrate in 5–15 min. Although gel extraction is necessary, we have shown that one round of the PS-Brick reaction from PCR and plasmid isolation to transformation can be performed in less than a single working day (Additional file 1: Fig. S2). A third distinctive characteristic of the PS-Brick scheme is its simplicity and convenience: sophisticated Golden Braid and MoClo frameworks required elaborate plasmid libraries to prepare donor DNA in the defined format [12, 32], which poses a barrier to their straightforward application and widespread adoption. PS-Brick only requires one acceptor vector with simple assembly rules, which can be easily mastered and applied by researchers.

The definition of technical standards for donor parts is the first step in implementing an idempotent assembly strategy [24]. The insertion parts of PS-Brick are prepared through direct PCR amplification, which adds appropriate adjacent RE site extensions to one of the primer pairs. PCR is generally recognized as the first and the foremost important tool in molecular biology and biotechnology [80]. A number of efficient and flexible PCR-based cloning methods have been developed, including OE PCR [44], In-Fusion BioBrick [29], QGA [14], SSEA [75],TAP [47] and 3G assembly [81]. PCR can be used to produce the desired DNA fragment in vitro and in large quantity from any form of available template DNA source, especially those stored in a community-wide collection. This means that the parts of a community-wide DNA assembly standard can easily be reused in PS-Brick assembly through PCR modification with newly synthesized primers, facilitating flexibility and universality in design and construction.

The need for elimination of internal restriction sites is still a general limitation for the RE-based strategies of DNA assembly. Compared with BioBrick methods with four given REs, PS-Brick can adopt two categories of REs, Type IIP (generating long stick overhangs) and Type IIS (generating blunt ends or one base overhangs). Hundreds of candidates for the Type IIP REs generate long sticky overhangs and four candidates (*Bmr*I, *Bci*VI, *Hph*I and *Mly*I) for the Type IIS REs used in PS-Brick assembly provide broad available options (Table 1). However, due to the limited number of commercial Type IIS endonucleases, it is often difficult to find an appropriate enzyme to avoid naturally occurring Type IIS sites within DNA parts. Thus, additional efforts, such as overlapping-PCR [44], directed mutagenesis, or direct DNA synthesis, will be needed to eliminate RE sites present in the DNA sequence prior to its use in the assembly system.

Combinatorial pathway engineering has been successfully used to produce various biochemicals [82, 83]. In a combinatorial strategy, multiple metabolic pathways with different expression levels are simultaneously assembled to generate a strain library. High-producing strains with balanced metabolic flux can be efficiently identified through one round of screening a strain library. However, PS-Brick is unfit for combinatorial pathway engineering because of its limitations in multi-part assembly. Golden Gate based methods such as Ecoflex [84], MoClo [33] and Start-Stop Assembly [42] can assemble more than 60 parts over two or three levels. In this study, the promotor PT and RBS were combined as one donor fragment (PT-BCD1) through overlap PCR, and the two genetic elements were assembled into an acceptor plasmid through one round of the PS-Brick reaction. In addition, five genetic fragments were synthesized as one donor DNA fragment Ptrc-BDC1-*kivD*-*ADH2*-Ter (Fig. 5b), and the five fragments were assembled into the acceptor plasmid through one round of the PS-Brick. Similarly, another multi-part assembly scheme of donor fragments was expected to be incorporated into the PS-Brick framework, generating improved technical features.

Golden Gate and its derivatives use only Type IIS REs cleaving outside of their recognition site, which allows for restriction and ligation to be performed in a one-pot reaction [30]. For the PS-Brick and BioBrick-like methods, the reactions of REs and T4 ligation were performed separately in different buffers and at different temperature. The assembly workflow of PS-Brick was more complex than that of the one-pot method.

Each common assembly method has significant advantages, but also limitations. Therefore, a combination of these methods could potentially fulfil the ideal goals while integrating their merits. For example, Golden Gate and Gibson assembly [81], multisite gateway recombination and Gibson Assembly [85] and multisite gateway cloning and homing endonucleases [86] have been successfully combined for developing novel hybrid technologies. PS-Brick provides a framework to assemble the recipient vector and the donor fragment DNA. Certain assembly schemes with one-pot or multi-part merits are expected to be incorporated into the PS-Brick scheme to generate a holistic framework.

## Conclusions

RE-assisted DNA assembly methods are widely used for their simple workflows, which can be easily mastered and applied by researchers. To the best of our knowledge, the PS-Brick framework is the first RE-assisted DNA assembly method using both Type IIP and IIS REs, which retains both the iterative strength of the IIP RE-assisted method and the seamless strength of the IIS RE-assisted method. We have developed an efficient and simple assembly method that simultaneously addresses these three highly desirable features. We assert that these characteristics will make PS-Brick a valuable addition to the toolbox of synthetic biologists and metabolic engineers.

## Methods

### Bacterial strains, plasmids and reagents

All bacterial strains and plasmids used in this study are listed in Additional file 1: Table S1. *E. coli* DH5α was used as a cloning host for PS-Brick assembly methods, and *E. coli* MG1655 was used for metabolic engineering. All primers used in this study were ordered from Thermo Fisher Scientific (Additional file 1: Table S2). The restriction enzyme *Bci*VI was purchased from Thermo Fisher Scientific, and all the other restriction enzymes and T4 DNA ligase were purchased from New England Biolabs (NEB) (Ipswich, MA, USA). KAPA Hot Start high-fidelity polymerase (Kapa Biosystems, Wilmington, MA) and Ex-Taq DNA Polymerase (TaKaRa-Bio, Japan) were used for amplification of donor parts. A DNA Purification Kit, Bacteria DNA Kit, Mini Plasmid Kit, RNAprep pure Kit and FastQuant RT Kit from TIANGEN BIOTECH were used for DNA preparation. GoTaq^®^ qPCR Master Mix (Promega, USA) was used for quantitative PCR. In addition, 2 × PCR Master Mix (TSINGKE, Beijing) was used for clone PCR. Tryptone and yeast extract were supplied from OXOID. Amino acids and antibiotics were acquired from Sigma-Aldrich. All other chemicals were purchased from Beijing Chemical Works unless otherwise stated.

### Construction of the original plasmids for PS-Brick

The plasmid pUC19 was used as the base vector for demonstration of the PS-Brick scheme. One *Bmr*I site and three *Mly*I sites located in the pUC19 vector backbones are removed through overlap extension PCR [44]. DNA fragments amplified with UC709-F/UC1179-R and UC1179-F/UC1695-R were fused by overlap PCR with primers UC709-F/UC1746-R (Additional file 1: Table S2) and then taken as the long primer for the pUC19 vector mutation. Another two *Bmr*I and *Mly*I sites present in the MCS sequence of the pUC19 vector were removed by *Sph*I and *Nde*I digestion. Two types of truncated mCherry fragments were amplified from the pSEVA237R vector [87] by the same forward primer mC-F flanked with an *Nde*I site, and a reverse primer mCB-R flanked by a *Sph*I/*Bmr*I site and another forward primer mCM-R flanked with *Sph*I/*Mly*I site, respectively. PCR products were column-purified and eluted with 35 μL of MilliQ grade water. The purified PCR product was digested with *Sph*I and *Nde*I and ligated with the same REs digested pUC19 vector backbones. After ligation and sequencing verification, the newly obtained vectors pOB and pOM from pUC19, containing the entrance sites *Sph*I/*Mly*I and *Sph*I/*Bmr*I, respectively, were taken as the original PS-Brick vectors for subsequent assembly. Primers UC1-6 listed in supplementary Table S2 were used for sequencing verification.

Three *Mly*I sites located in the pACYC184 vector backbones were mutated through overlap extension PCR with primers AC3211-F/AC727-R and AC727-F/AC1143-R. Another *Mly*I site located in the MCS sequence of the pACYC184 vector was removed by *Hind*III and *Nru*I digestion. The remaining pACYC184 vector backbone without the *Mly*I site was linked with the PCR products of truncated *thrABC* operon genes by primers TAB-F/TAB-R, TBC-F/TBC-R and TC-F/TC-R. A NruI site was designed in the primer outside of the terminator of the *thrC* gene, and a *Hind*III site was designed outside of the truncated *thrA* and internally adjacent to the *Mly*I site. After ligation and sequencing verification, the newly obtained vector pOthr containing adjacent HindIII/MlyI entrance sites was used for subsequent metabolic pathway assembly.

### The workflow of PS-Brick assembly

#### PS-Brick assembly with *Sph*I/*Mly*I entrance site

The primer FM-R flanked by the *Sph*I/*Mly*I site and the primer FM-F were used to amplify the donor fragment FM by KAPA Hot Start high-fidelity polymerase. PCR procedures with KAPA Hot Start high-fidelity polymerase consisted of one cycle of 95 °C for 3 min, 27 cycles of 98 °C for 20 s, 65 °C for 20 s and 72 °C for 30 s and one cycle of 72 °C for 1 min. The PCR product was run out on a 1% agarose gel stained with GelSafe (TSINGKE, Beijing), and amplicons of interest were column-purified and eluted with 35 μL of MilliQ grade water. The purified PCR products were digested with SphI only for 30 min, heat-inactivated at 60 °C for 20 min and then column-purified for ligation.

The original PS-Brick vector pOM containing the *Sph*I/*Mly*I entrance site was cleaved by *Sph*I and *Mly*I for 30 min in the same buffer. All RE-digestion reactions were performed at 37 °C in a 50-µL volume containing 20 units of enzyme and 1 µg of DNA. The double-digested vectors were heat-inactivated at 60 °C for 20 min and then column-purified for ligation with the *Sph*I-digested PCR fragments. The DNA concentration was determined by a Nanodrop 2000c (Thermo Fisher Scientific, Waltham, MA).

Aligation reaction mix of 10 μL, containing 1 μL of T4 DNA ligase, 20 ng of linearized vector and fivefold molar excess of insert DNA, was incubated at 25 °C for 15 min and chilled on ice and then transformed into 100 μL of *E. coli* DH5α competent cells. Finally, the mixture was spread onto a selection plate and incubated overnight at 37 °C. The colonies were verified by DNA sequencing, and the correct plasmids were used as the acceptor vector for the next round of PS-Brick.

#### PS-Brick assembly with *Hind*III/*Mly*I entrance site

Except for the use of the Type IIP RE *Hind*III and the original vector pOthr, all the conditions were same as above.

#### PS-Brick assembly with *SphI*/*Bmr*I and *Hind*III/*Bci*VI entrance sites

The primers flanked with *Sph*I/*Bmr*I or *Hind*III/*Bci*VI sites were used to amplify the donor fragment by Ex-Taq DNA Polymerase. PCR procedures with Ex-Taq Polymerase were set as follows: one cycle of 94 °C for 5 min, 27 cycles of 94 °C for 30 s, 54 °C for 30 s and 72 °C for 30 s and a final extension of 5 min. All the other conditions were the same as above except for the REs and the original vectors.

#### CRISPR–Cas9 genome editing

The editing process was performed as previously described [70]. In short, MG1655 competent cells harbouring pCas9 were prepared, and arabinose (10 mM final concentration) was added to the culture for λ-Red induction. For electroporation, 50 μL of cells was mixed with 200 ng of ptargetET-*tdh*-*ilvA*. Electroporation was performed in a 2-mm Gene Pulser cuvette (Bio-Rad) at 2.5 kV and the product was suspended immediately in 1 mL of LB medium. Cells were recovered at 30 °C for 1 h before being spread onto LB agar containing kanamycin (50 mg/L) and spectinomycin (50 mg/L) and incubated overnight at 30 °C. Transformants were identified by colony PCR and DNA sequencing with primer pairs *ilvA*-I-F/R and *tdh*-I-F/R for *ilvA* and *tdh*, respectively. For the curing of ptargetET-*tdh*-*ilvA*, the edited colony was inoculated into 2 mL of LB medium containing kanamycin (50 mg/L) and IPTG (isopropyl-D-thiogalactopyranoside; 0.5 mmol/L). The culture was incubated for 8 to 16 h, diluted and spread onto LB plates containing kanamycin (50 mg/L). The colonies were confirmed as cured by determining their sensitivity to spectinomycin (50 mg/L). pCas was cured by non-selectively growing the colonies overnight at 37 °C.

### Media, culture conditions and fed-batch fermentation in the bioreactor

Lysogeny Broth (LB) was used during cloning work, consisting of tryptone 10 g/L, yeast extract 5 g/L and NaCl 10 g/L. The composition of solid culture medium, Lysogeny Agar (LA), was identical, except for the addition of 15 g/L agar. If required, media were supplemented with the antibiotics ampicillin (100 μg/mL), kanamycin (50 μg/mL) or chloramphenicol (34 μg/mL).

Shake-flask fermentation medium: MOPS 80 g/L, glucose 20 g/L, (NH4)_2_SO_4_ 20 g/L, KH_2_PO_4_ 2 g/L, MgSO_4_·7H_2_O 2 g/L, yeast extract 4 g, betaine 2 g, trace element solution 5 mL.

Seed medium for fed-batch fermentation [69]: glucose 40 g/L, (NH_4_)_2_SO_4_ 15 g/L, KH_2_PO_4_ 2 g/L, MgSO_4_·7H_2_O 2 g/L, yeast extract 2 g/L, l-isoleucine 0.1 g/L, CaCO_3_ 10 g/L, trace element solution 5 mL/L.

Fed-batch fermentation medium [69]: glucose 10 g/L, (NH_4_)_2_SO_4_ 10 g/L, KH_2_PO_4_ 2 g/L, MgSO_4_·7H_2_O 2 g/L, yeast extract 2 g/L, trace element solution 10 mL/L. The trace element solution (per litre): FeSO_4_·7H_2_O 6 g, CaCl_2_ 1.35 g, ZnSO_4_·7H_2_O 0.8 g, MnSO_4_·4H_2_O 1.5 g, CuSO_4_·5H_2_O 0.15 g, (NH_4_)_6_Mo_7_O_24_·4H_2_O 0.2 g, H_3_BO_3_ 0.1 g, CoCl_2_·6H_2_O 0.25 g, 35% HCl 10 mL.

Strains cultured on LB plates were incubated at 37 °C for 12 h. For preculture, one loop of cells was inoculated into a tube with 3 mL of LB medium and incubated for 8 h at 37 °C with shaking at 220 rpm on a rotary shaker. For the seed culture, 1.0 mL of the obtained preculture broth was inoculated into 30 mL of seed culture with the same culture conditions for 8 h. Two copies of the obtained seed culture were then inoculated into 2 L of fermentation medium for fed-batch culture.

Fed-batch fermentation was performed in a BioFlo^®^ 115 Fermentor System (New Brunswick Scientific, Edison, NJ, USA) consisting of 7.5-L double-jacketed glass vessels with a working volume of 2–3 L. Data logging and operational parameters were controlled by the BioCommand Plus BioProcessing Software (New Brunswick Scientific). The temperature was maintained at 37 °C, and the pH was maintained at 6.9 by addition of 25% ammonia. Dissolved oxygen tension was maintained at 50% of air saturation by automatically cascading with stirrer speeds ranging from 200 to 1000 rpm. Pure oxygen was mixed with air to afford sufficient dissolved oxygen when the stirrer speed exceeded 1000 rpm. A silicone-based antifoaming agent was added as required. The concentration of glucose over all of the fed-batch cultures was maintained within the range of 10 ± 5 g/L by supplying 700 g/L of glucose reservoir. The continuous feeding rate of the glucose reservoir was regulated according to the residual glucose concentration. For isoleucine auxotroph strains, isoleucine was continuously supplied according to our previous report [69].

### Preparation and transformation of chemically competent E. coli cells

For the preparation of chemically competent* E. coli* cells, inoculated a single colony into 5 mL of LB medium with or without appropriate antibiotics and rotated the culture overnight at 37 °C. Then, used 1% overnight culture to inoculate 50 mL of LB medium and incubated at 37 °C until the absorbance at 600 nm was between 0.4 and 0.5. Spun the cell suspension for 10 min at 6000 rpm, discarded the supernatant and gently resuspended the pellet in 20 mL ice-cold buffer solution I (including 1.33 g of CaCl_2_, 30 mL of glycerine and 142 mL of MilliQ grade water). After incubation on ice for 30 min, cells were spun down at 4000 rpm for 5 min and being gently resuspended in 2 mL of buffer solution I. Distributed the cell suspension in 100 μL aliquots in 0.2 mL microfuge tubes and stored the tubes at − 80 °C.

For transformation, a tube of 100 μL chemically competent *E. coli* cells was thawed on ice. 50 ng plasmid DNA was added to the cell mixture and being mixed carefully with cells. The mixture was then placed on ice for 30 min, heat shock treated at 42 °C for 90 s and chilled on ice for another 3 min. 900 μL LB was added to the tube and the cells were recovered at 37 °C with shake vigorously for 1 h. Finally, 50–100 μL mixture was spread onto selection plate and incubated overnight at 37 °C.

### Analytical methods

The biomass concentration was monitored by measuring the optical density at 600 nm (OD_600_). Dry cell weight (DCW) was calculated on the basis of OD_600_ (1 OD_600_ = 0.42 g DCW/L) [69].

Quantitative PCR was performed using GoTaq qPCR master mix (Promega, USA) in a 20-μL mixture with a LightCycler^®^ 96 RealTime PCR System (Roche, Switzerland) according to our previous report [88].

Fermentation samples were centrifuged at 8000g for 5 min, and the supernatants were used for analysis of the substrate and product concentrations. The concentration of glucose was assayed with an enzyme electrode analyser (SBA-40D; Institute of Biology, Shandong, China) containing glucose oxidase.

Amino acids were quantified by an HPLC (1200 series; Agilent Technologies, USA) equipped with an Eclipse XDB-C18 column (4.6 mm × 150 mm; Agilent Technologies, USA). UV absorption was performed at 360 nm. Samples were pre-column derivatized with 2,4-dinitrofluorobenzene as the derivatization reagent. The gradient mobile phase was set as in our previous report [89]. In addition, 2-ketobutyrate was quantified by an HPLC equipped with a Zorbax SB-Aq column (4.6 × 250 mm; Agilent Technologies, USA), and 20 mM KH2PO4 (pH 2.2) was used as a mobile phase with a flow rate of 0.5 mL/min. UV absorption was measured at 210 nm. The 1-propanol was analysed using a Gas Chromatograph Mass Spectrometer (GCMSQP2010 Ultra, Shimadzu, Japan) connected to an AOC-20i Auto-sample using a TG-WAXMS (length: 30 m; I.D.: 0.25 mm; film: 0.25 μm) (Thermo Scientific, USA). The samples were directly diluted 1:10 with methanol and the operating set up followed as the previous report [73]. The concentration was determined according to a calibration curve with an external standard. The peaks were identified by retention time and quantified using the intensity of the peak at one specific m/z value according to our previous report [90].

## Additional file

**Additional file 1: Figure S1.** The cutting efficiency of *Bmr*I (A) and *Mly*I (B) on vector pOB and pOM was tested through electrophoresis with cutting time ranging from 15 min to 180 min.** Figure S2.** The workflow of PS-Brick assembly. The insertion part F1 was PCR product amplified by Ex-Taq DNA Polymerase or KAPA high-fidelity polymerase. The PCR product was gel-purified, digested with IIP RE only and then column-purified for ligation. The base plasmids containing entrance REs site were firstly cleaved by Type IIS RE for 15 min, and the linearized vectors were separated by electrophoresis, gel purified, and then recovered for the second digestion by Type IIP RE for 15 min. The double REs-digested vectors and Type IIP RE-digested PCR products were heat-inactivated at 60°C for 20 min and then were column-purified for ligation together. After ligation reaction for 15 min, the ligation mix was transformed into *E. coli* DH5α competent cells. The chemically competent *E. coli* cells mixed with DNA were then placed on ice for 30 min, heat shock at 42°C for 90 s, chilled on ice for another 3 min, incubated at 37°C for 1 h. Finally, the mixture was spread onto selection plate and incubated overnight at 37 °C.** Figure S3.** Transcriptional analysis of the *ppc*, *aspA*, *aspC*,* asd* and *pntAB* genes by real-time RT-PCR. The control strain was *E. coli* K12 MG1655/ pACYC184-*thrA*^*433phe*^*BC*, and the engineering strains were *E. coli* K12 MG1655 strain harboring plasmids pACYC184-*thrA*^*433phe*^*BC-ppc/aspA/aspC/asd/pntAB*, respectively. Data shown are mean values from three biological replicates, and the standard deviations are presented. Symbol “*” denotes the relative expression level of the gene overexpressed in plasmid. Take Panel A, quantitative PCR of *ppc* gene for all the strains, as an example, “*” denotes the strain overexpressed *ppc* gene, and the relative expression level of *ppc* was predictively higher than other strains.** Figure S4.** Transcriptional analysis of the *ppc*, *aspA*, *aspC*,* asd* and *pntAB* genes by real-time RT-PCR. The control strain was *E. coli* K12 MG1655/ pACYC184-*thrA*^*433phe*^*BC-asd*, and the engineering strains were *E. coli* K12 MG1655 strain harboring plasmids pACYC184-*thrA*^*433phe*^*BC-asd-rhtA/rhtB/rhtC/yeaS*, respectively. Data shown are mean values from three biological replicates, and the standard deviations are presented. “*” denotes the relative expression level of the gene overexpressed in plasmid. Take Panel A, quantitative PCR of *rhtA* gene for all the strains, as an example, “*” denotes the strain overexpressed *rhtA* gene, and the relative expression level of *rhtA* was predictively higher than other strains.** Figure S5.** Construction of CRISPR arrays containing sequence repeats via TA clone/BciVI based PS-Brick assembly. The N20 sequence flanked with the same promoter pJ23119 and the sgRNA sequence located in pTargetET vector. The entrance site of *Hind*III/*Bci*VI and editing template was introduced into pTargetF to generate original vector ptargetET for PS-Brick assembly. The N20 fragments of *tdh*, *ilvA* and *tdcC* fixed with the same promoter and sgRNA at each end were sequentially inserted into ptargetET through three rounds of PS-Brick reactions.** Table S1.** Strains and plasmids used in this study with relevant characteristics.

## Data Availability

All data generated or analysed during this study are included in this published article (and its additional file).

## Abbreviations

RE: restriction endonucleases
DBTL: design–build–test–learn
iGEM: international Genetic Engineered Machine
CFUs: colony-forming units
CRISPR: clustered regularly interspaced short palindromic repeat
sgRNA: single-guide RNA
bp: base pairs
*E. coli*: *Escherichia coli*
HPLC: high-performance liquid chromatography
WT: wild-type
NADPH and NADP: reduced and oxidized form of nicotinamide adenine dinucleotide phosphate, respectively
OD_600_: optical density at wavelength (*λ*) 600 nm
