# Supplementary material for: A seamless and iterative DNA assembly method named PS-Brick and its assisted metabolic engineering for threonine and 1-propanol production
Source: Biotechnol Biofuels. 2019 Jul 15;12:180. doi: 10.1186/s13068-019-1520-x (PMC6628500; doi:10.1186/s13068-019-1520-x)
Supplement: Supplementary file 1 — Additional file 1: Figure S1. The cutting efficiency of BmrI (A) and MlyI (B) on vector pOB and pOM was tested through electrophoresis with cutting time ranging from 15 min to 180 min. Figure S2. The workflow of PS-Brick assembly. The insertion part F1 was PCR product amplified by Ex-Taq DNA Polymerase or KAPA high-fidelity polymerase. The PCR product was gel-purified, digested with IIP RE only and then column-purified for ligation. The base plasmids containing entrance REs site were firstly cleaved by Type IIS RE for 15 min, and the linearized vectors were separated by electrophoresis, gel purified, and then recovered for the second digestion by Type IIP RE for 15 min. The double REs-digested vectors and Type IIP RE-digested PCR products were heat-inactivated at 60°C for 20 min and then were column-purified for ligation together. After ligation reaction for 15 min, the ligation mix was transformed into E. coli DH5α competent cells. The chemically competent E. coli cells mixed with DNA were then placed on ice for 30 min, heat shock at 42°C for 90 s, chilled on ice for another 3 min, incubated at 37°C for 1 h. Finally, the mixture was spread onto selection plate and incubated overnight at 37 °C. Figure S3. Transcriptional analysis of the ppc, aspA, aspC, asd and pntAB genes by real-time RT-PCR. The control strain was E. coli K12 MG1655/ pACYC184-thrA433pheBC, and the engineering strains were E. coli K12 MG1655 strain harboring plasmids pACYC184-thrA433pheBC-ppc/aspA/aspC/asd/pntAB, respectively. Data shown are mean values from three biological replicates, and the standard deviations are presented. Symbol “*” denotes the relative expression level of the gene overexpressed in plasmid. Take Panel A, quantitative PCR of ppc gene for all the strains, as an example, “*” denotes the strain overexpressed ppc gene, and the relative expression level of ppc was predictively higher than other strains. Figure S4. Transcriptional analysis of the ppc, aspA, aspC, asd and pntAB gene [file 13068_2019_1520_MOESM1_ESM.docx]

Additional Information

For

**A seamless and iterative DNA assembly method named PS-Brick and its assisted metabolic engineering for threonine and 1-propanol production**

Shuwen Liu ^a,1^, Haihan Xiao ^a,b,1^, Fangfang Zhang ^a,c^, Zheng Lu ^a^, Yun Zhang ^a^, Aihua Deng ^a^, Tingyi Wen ^a,d,*^

^a^CAS Key Laboratory of Pathogenic Microbiology and Immunology, Institute of Microbiology, Chinese Academy of Sciences, Beijing 100101, China

^b^University of Chinese Academy of Sciences, Beijing 100049, China

^c^Institute of Physical Science and Information Technology, Anhui University, Hefei 230039, China

^d^Savaid Medical School, University of Chinese Academy of Sciences, Beijing 100049, China

* Corresponding authors: Tel: 86-10-64806119; Fax: 86-10-64806157; Email: wenty@im.ac.cn

^1^ These authors contributed equally to the paper as first authors.

**Methods**

**Iterative PS-Brick assembly for DBTL cycles of metabolic engineering**

The insert PCR products of *thrA* were amplified by Kapa Hot start high-fidelity polymerase using one forward primer TA-F flanked with the adjacent *Hind*III/*Mly*I site, and respective 20 reverse primers TA^Amino Acid^-R generating codon saturation mutagenesis in the 433th residue of ThrA (Additional file 1: Table S2). 20 PCR insertions were digested by *Hind*III and separately ligated with *Hind*III/*Mly*I digested pOthr, generating 20 vectors pthr*A^433^BC* with the same adjacent *Hind*III/*Mly*I site for next round of parts incorporation. Internal *Hind*III and *Mly*I restriction sites in sequence were mutated off [1] if required (Additional file 1: Table S2). The genome of *E. coli* MG1655 provided the PCR template for all genes. All the REs digestion, purification, T4 ligation, and transformation of PS-Brick reaction were performed similarly to the previous section.

Similarly, PCR products of *aspA* amplified with primers aspA-F/aspA-R, *aspC* with primers aspC1-F/aspC1-R and aspC2-F/aspC2-R; *ppc* with primers ppc1-F/ppc1-R, ppc2-F/ppc2-R, ppc3-F/ppc3-R, and ppc4-F/ppc5-R; *asd* with primer asd1-F/asd1-R and asd2-F/asd2-R; *pntAB* with primer pnt1-F/pnt1-R, pnt2-F/pnt2-R, pnt3-F/pnt3-R, pnt4-F/pnt4-R and pnt5-F/pnt5-R (Additional file 1: Table S2), were inserted into pthr*A^433phe^BC* through the second round of PS-Brick reaction, respectively. Through the third round of PS-Brick assembly, PCR products of *rhtA* amplified with primers rhtA-F/rhtA-R, *rhtB* with primers rhtB-F/rhtB-R, *rhtC* with primers rhtC-F/rhtC-R and *yecC* with primers yec-F/yec-R were incorporated into pthr*A^433phe^BC*-*asd*, and subsequently elements P_T_ and BCD1 were fused by overlap PCR with primers T-F/TBCD-R and TBCD-F/BCD-R, and then inserted in front of the five export genes, respectively, via the fourth round of PS-Brick assembly.

The donor DNA fragment Ptrc-BDC1-kivD-ADH2-Ter (Fig. 5b), containing Promoter Ptrc (ttgacaattaatcatccg gctcgtataatgt), BCD1[2], *kivD* (Accession Number AJ746364), *ADH2* of *Saccharomyces cerevisiae* and Terminator (CTAGCATAACCCCTTGGGGCCTCTAAACGGGTCTTGAGGGG TTTTTTG), was synthesized by Genscript Biotech. Corp. (Nanjing, China). The stop codon UAA in *kivD* was in front of start codon ATG in *ADH2*. The internal *Hind*III and *Mly*I restriction sites in sequence were removed through DNA synthesis. PCR products of Ptrc-BDC1-kivD-ADH2-Ter amplified with primers Prop-F/ Prop-R (Additional file 1: Table S2) were incorporated into pthrA433pheBC-asd via one round of PS-Brick assembly.

**Construction of CRISPR-array plasmids and genome editing**

Previously reported CRISPR-Cas9 genome editing system containing vectors pCas9 and pTargetF were applied for *ilvA* and *tdh* deletion [3]. The *Bci*VI site of pTargetF was mutated through PCR using primer pair TGB-F/R, DpnI digestion and transformation. The donor DNA had a 500-bp sequence homologous to each side (upstream or downstream) of the *tdcC, ilvA* and *tdh* loci in the genome, and was used as the genome editing template. The editing templates of the *tdcC*, *ilvA* and *tdh* were combined together through overlap PCR amplified by primers tdc1/2-F/R, ilv1/2-F/R and tdh1/2-F/R. The entrance restriction sites of adjacent *Hind*III-*Bci*VI were designed in the extensions of primer tdc1-F, and *Bam*HI site was added in the extensions of primer tdh2-R. The overlap PCR fragments and pTargetF vectors [3] were double digested by *Bam*HI and *Hind*III. After ligation and sequencing verification, the newly obtained vector ptargetET containing adjacent *Hind*III-*Bci*VI site was used for subsequent assembly of sgRNA arrays with the same promoter and terminator. Firstly, two pTargetF vectors containing targeting N20 sequence of *tdh* and *ilvA*, respectively, were constructed through PCR with primers N20-tdh-F/R and N20-ilvA-F/R including the modified N20 sequence and followed by DpnI digestion. Secondly, the sgRNA fragment was amplified from pTargetF-tdh by EX Taq DNA polymerase with primers sgRNA-F/R, and was then digested by *Hind*III and inserted into the *Hind*III/*Bci*VI-digested ptargetET, resulting in ptargetET-*tdh* also containing adjacent *Hind*III-*Bci*VI sites that designed in the PCR primer sgRNA-F. Thirdly, the sgRNA fragment was amplified from pTargetF-*ilvA* by the same primers sgRNA-F/R, and subsequently digested by *Hind*III and then ligated with the *Hind*III/*Bci*VI-digested ptargetET-*tdh*, resulting in ptargetET-*tdh*-*ilvA* consisting of double sgRNAs. The correct ptargetET -sgRNAs ligations were identified by colony PCR and DNA sequencing using the primer pair TG-F/sgRNA-R.


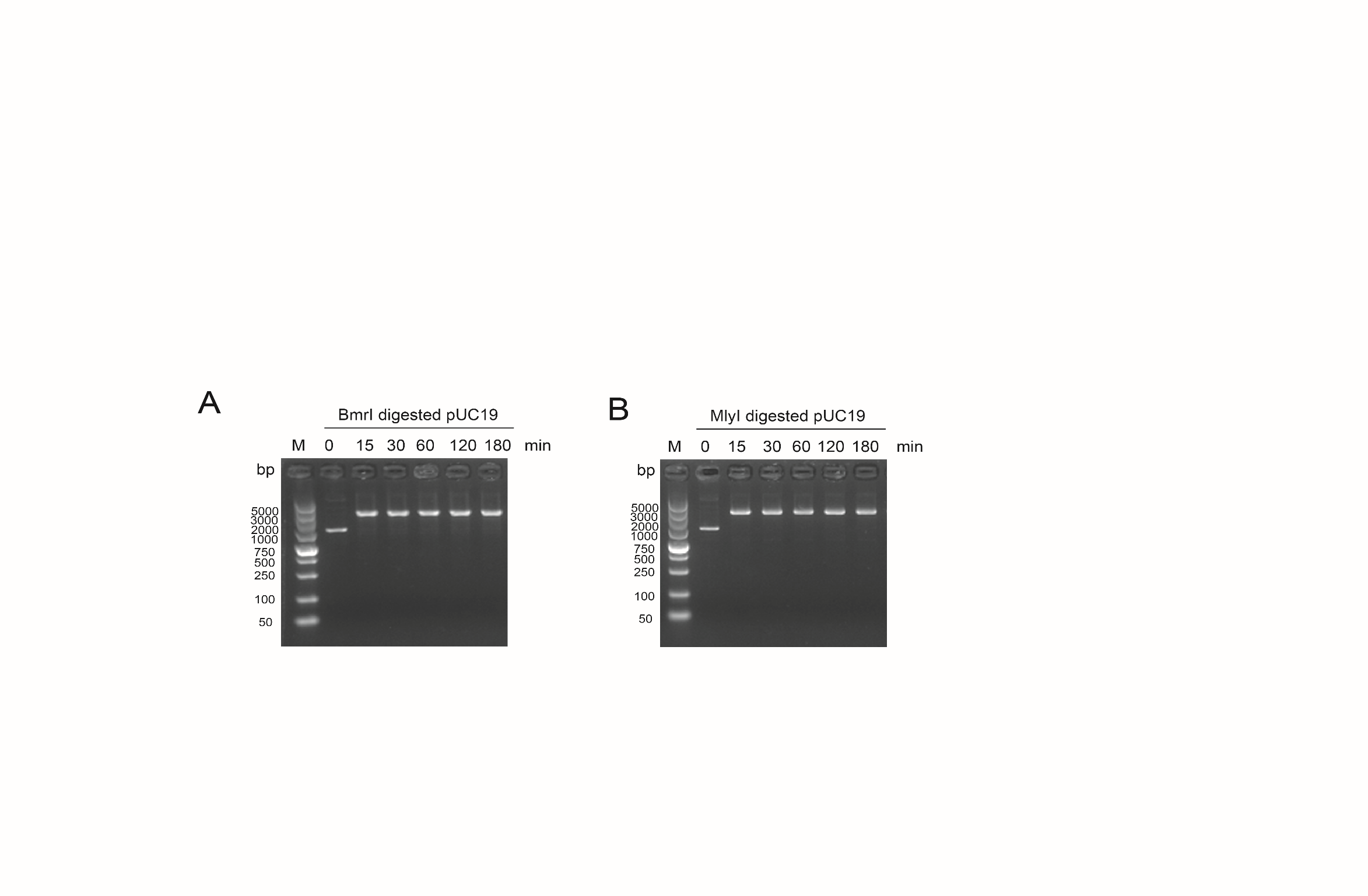


Fig. S1. The cutting efficiency of BmrI (A) and *Mly*I (B) on vector pOB and pOM was tested through electrophoresis with cutting time ranging from 15 min to 180 min.


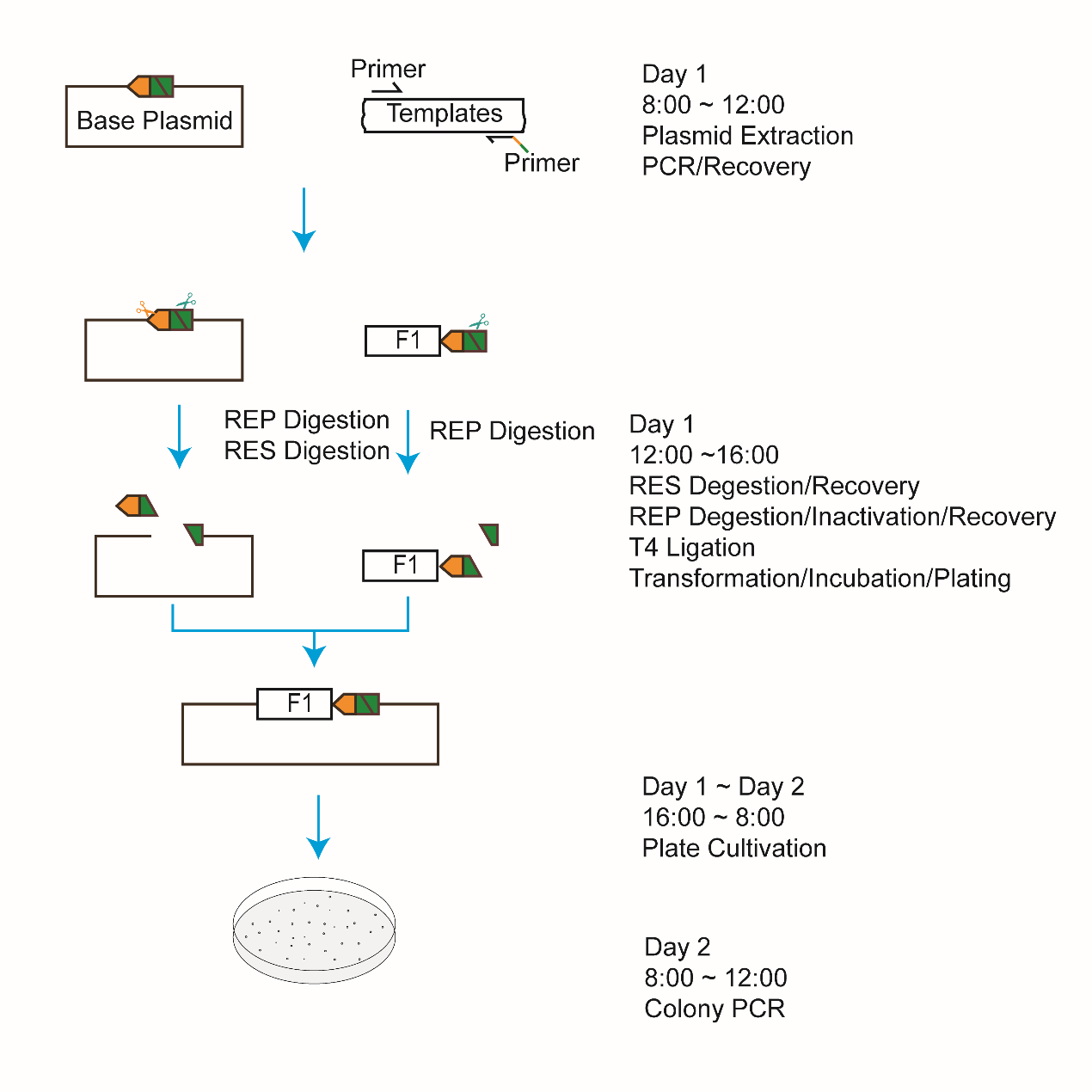


Fig. S2. The workﬂow of PS-Brick assembly. The insertion part F1 was PCR product amplified by Ex-Taq DNA Polymerase or KAPA high-fidelity polymerase. The PCR product was gel-purified, digested with IIP RE only and then column-purified for ligation. The base plasmids containing entrance REs site were firstly cleaved by Type IIS RE for 15 min, and the linearized vectors were separated by electrophoresis, gel purified, and then recovered for the second digestion by Type IIP RE for 15 min. The double REs-digested vectors and Type IIP RE-digested PCR products were heat-inactivated at 60°C for 20 min and then were column-purified for ligation together. After ligation reaction for 15 minutes, the ligation mix was transformed into *E. coli* DH5α competent cells. The chemically competent *E. coli* cells mixed with DNA were then placed on ice for 30 min, heat shock at 42°C for 90 s, chilled on ice for another 3 min, incubated at 37°C for 1 h. Finally, the mixture was spread onto selection plate and incubated overnight at 37 °C.


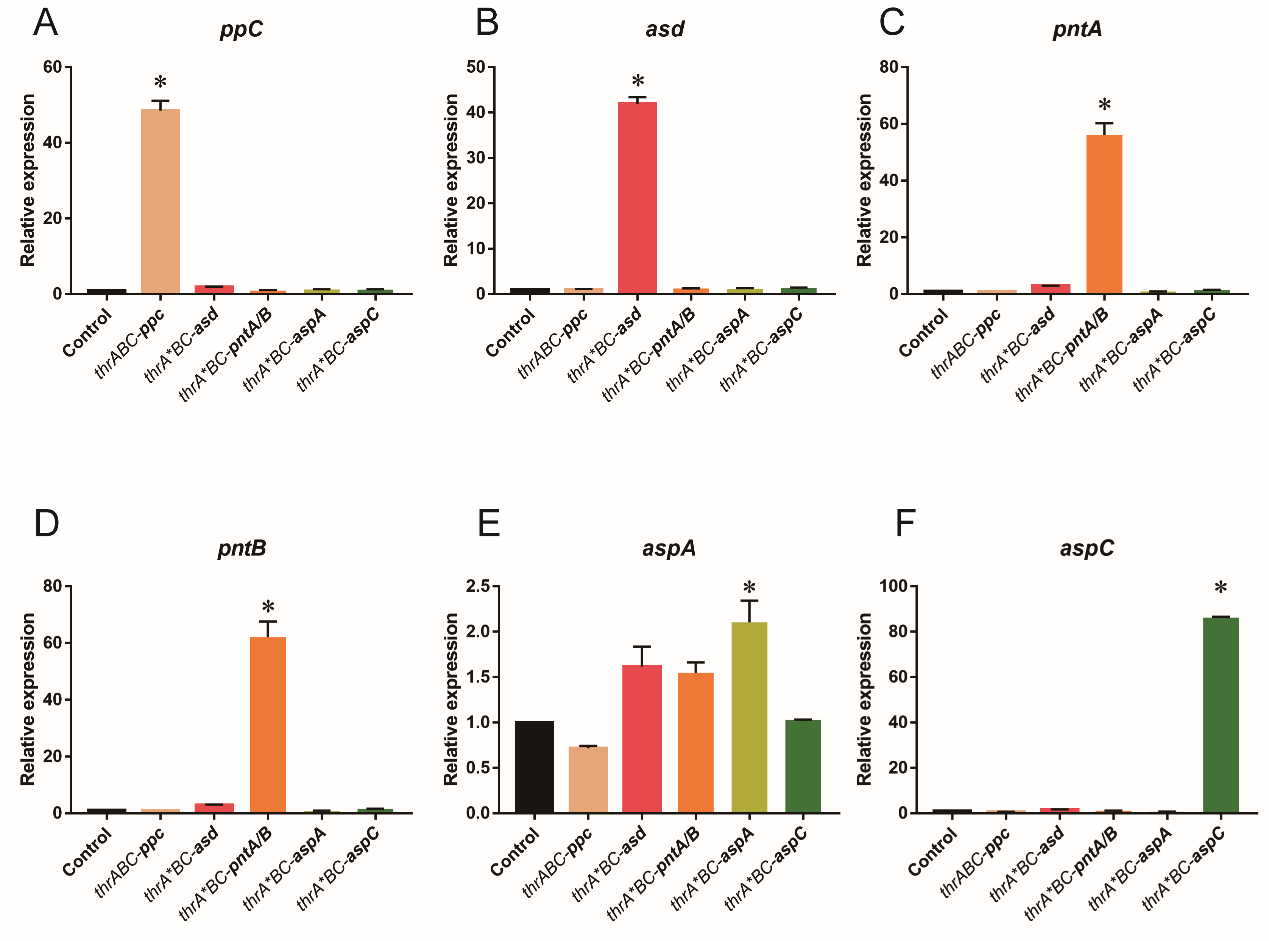


Fig. S3. Transcriptional analysis of the *ppc*, *aspA*, *aspC*, *asd* and *pntAB* genes by real-time RT-PCR. The control strain was *E. coli* K12 MG1655/ pACYC184-*thrA^433phe^BC,* and the engineering strains were *E. coli* K12 MG1655 strain harboring plasmids pACYC184-*thrA^433phe^BC-ppc/aspA/aspC/asd/pntAB*, respectively. Data shown are mean values from three biological replicates, and the standard deviations are presented. Symbol “*” denotes the relative expression level of the gene overexpressed in plasmid. Take Panel A, quantitative PCR of *ppc* gene for all the strains, as an example, “*” denotes the strain overexpressed *ppc* gene, and the relative expression level of *ppc* was predictively higher than other strains.


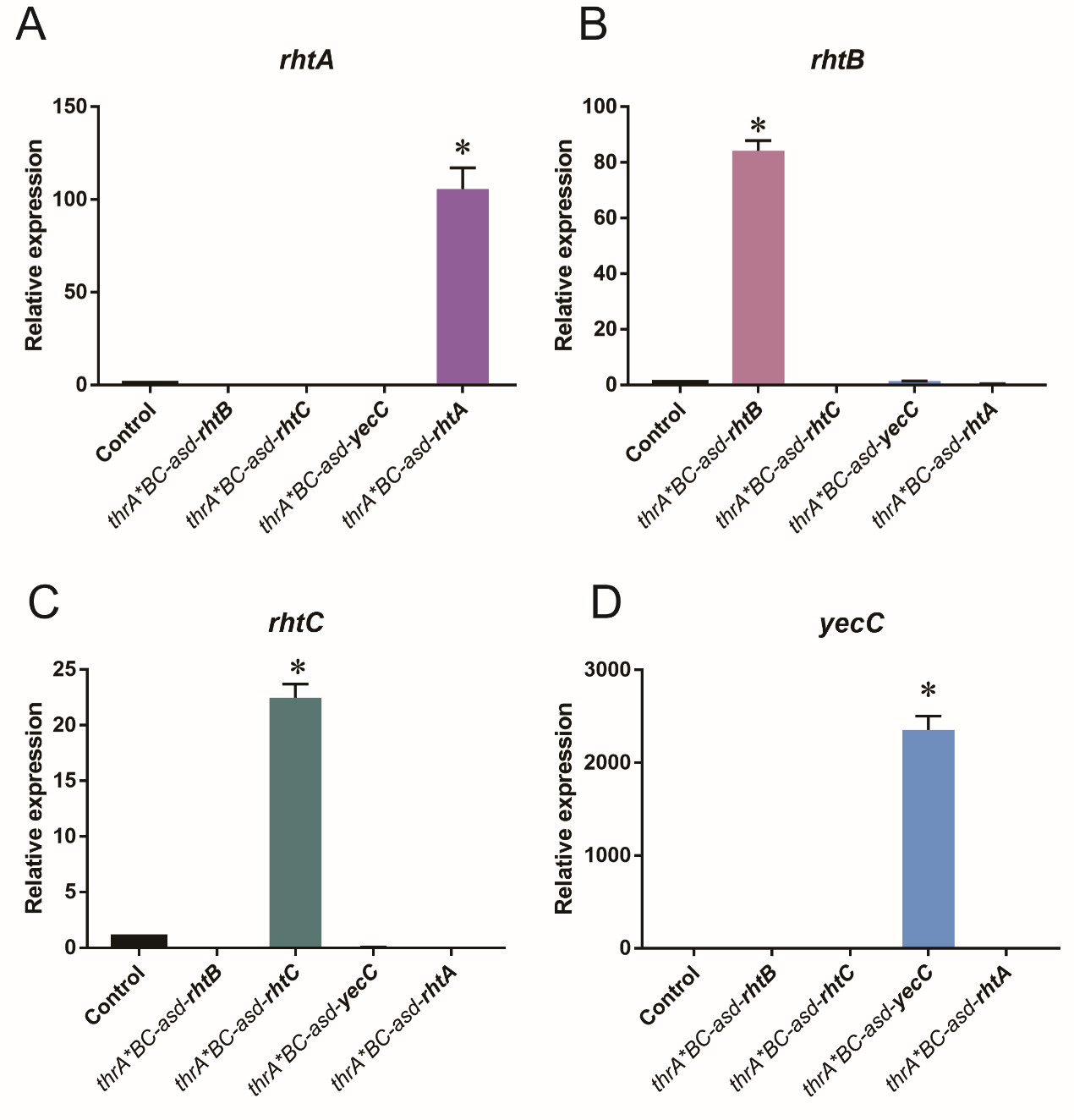


Fig. S4. Transcriptional analysis of the *ppc*, *aspA*, *aspC*, *asd* and *pntAB* genes by real-time RT-PCR. The control strain was *E. coli* K12 MG1655/ pACYC184-*thrA^433phe^BC-asd*, and the engineering strains were *E. coli* K12 MG1655 strain harboring plasmids pACYC184-*thrA^433phe^BC-asd-rhtA/rhtB/rhtC/yeaS*, respectively. Data shown are mean values from three biological replicates, and the standard deviations are presented. * denotes the relative expression level of the gene overexpressed in plasmid. Take Panel A, quantitative PCR of *rhtA* gene for all the strains, as an example, “*” denotes the strain overexpressed *rhtA* gene, and the relative expression level of *rhtA* was predictively higher than other strains.


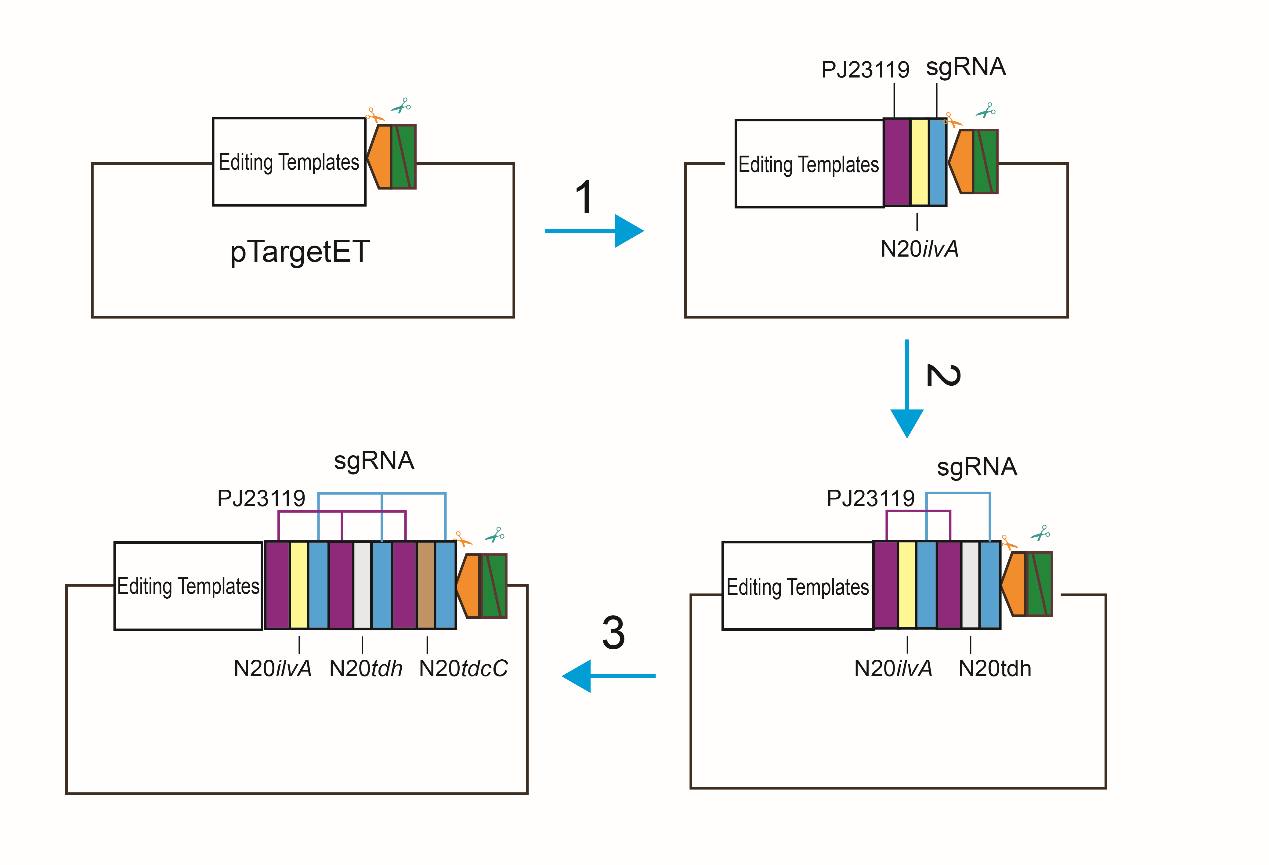


Fig. S5. Construction of CRISPR arrays containing sequence repeats via TA clone/*Bci*VI based PS-Brick assembly. The N20 sequence flanked with the same promoter pJ23119 and the sgRNA sequence located in pTargetET vector. The entrance site of *Hind*III/*Bci*VI and editing template was introduced into pTargetF to generate original vector ptargetET for PS-Brick assembly. The N20 fragments of *tdh*, *ilvA* and *tdcC* fixed with the same promoter and sgRNA at each end were sequentially inserted into ptargetET through three rounds of PS-Brick reactions.

Table S1 Strains and plasmids used in this study with relevant characteristics.

| **Strains and plasmids** | **Relevant characteristics** | **Sources** |
| --- | --- | --- |
| **Strains** | | |
| *E. coli* DH5α | F^−^ *endA1 glnV44 thi-1 recA1 relA1 gyrA96 deoR nupG* ϕ80d*lacZ*ΔM15 Δ(*lacZYA-argF*)*U169 hsdR17* (r_K_^−^ m_K_^+^) λ^−^ | Invitrogen |
| *E. coli* MG1655 | K-12; F^−^ λ^−^ *rph-1* | ATCC #700926 |
| MG1655Δ2 | MG1655Δ*ilvA*Δ*tdh* | This study |
| **Plasmids** | | |
| pUC19 | Vector backbone, pMB1 ori Amp^r^ | TaKaRa #D3219 |
| pO19 | pUC19 with three mutated *Bci*VI sites and one mutated BmrI site | This study |
| pOB | pO19 carrying truncated *mCherry* gene segment with SphI/BmrI entrance site | This study |
| pOM | pUC19 carrying truncated *mCherry* gene segment with SphI/*Mly*I entrance site | This study |
| pACYC184 | Vector backbone, p15A ori Cm^r^ | New England Biolabs |
| pO184 | pACYC184^718T/A,1150A/T,3219A/T^ | This study |
| pOthr | pO184 carrying truncated *thrABC* genes with adjacent *Hind*III/*Mly*I entrance site | This study |
| pthrA^433^BC series | pOthr carrying partial *thrA** encoding gene with 20 different codon saturation mutagenesis (Phe：TTT, Leu:CTG, Ile:ATT, Met:ATG, Val:GTG, Ser:AGC, Pro:CCG, Thr:ACC, Ala:GCG, Tyr:TAT, His:CAT, Gln:CAG, Asn:AAC, Lys:AAA, Asp:GAT, Glu:GAA, Cys:TGC, Trp:TGG, Arg:AGA, Gly:CGT) in the 433th residue | This study |
| p*thrA^433phe^BC*–*aspA* | p*thrA^433phe^BC* carrying *aspA* gene | This study |
| p*thrA^433phe^BC*–*aspC* | p*thrA^433phe^BC* carrying *aspC* gene | This study |
| p*thrA^433phe^BC*–*ppc* | p*thrA^433phe^BC* carrying *ppc* gene | This study |
| p*thrA^433phe^BC*–*asd* | p*thrA^433phe^BC* carrying *asd* gene | This study |
| p*thrA^433phe^BC*–*pntA/B* | p*thrA^433phe^BC* carrying *pntAB* gene | This study |
| p*thrA^433phe^BC*–*asd–rhtA* | p*thrA^433phe^BC*–*asd* carrying *rhtA* coding sequence with PT promoter and BCD1 | This study |
| p*thrA^433phe^BC*–*asd–rhtB* | p*thrA^433phe^BC*–*asd* carrying *rhtB* coding sequence with PT promoter and BCD1 | This study |
| p*thrA^433phe^BC*–*asd–rhtC* | p*thrA^433phe^B*C–*asd* carrying *rhtC* coding sequence with PT promoter and BCD1 | This study |
| p*thrA^433phe^BC*–*asd–yecC* | p*thrA^433phe^BC*–*asd* carrying *yecC* coding sequence with PT promoter and BCD1 | This study |
| pCas | repA101(Ts) kan Pcas-cas9 ParaB-Red lacIq Ptrc-sgRNA-Pmb1 | [4] |
| pTargetF | vector backbone for expressing sgRNA, *Pmb1ori* *Amp^r^*^,^ | [4] |
| pTargetF-*tdh* | pTargetF carrying sgRNA with an N20 sequence for targeting the *tdh* locus, N20+PAM: CCGTGCGGTTAACGTCGCCAAA | This study |
| pTargetF-*ilvA* | pTargetF carrying sgRNA with an N20 sequence for targeting the ilvA locus, N20+PAM: CTTCATCAAAGTTCGCGCCGTGG | This study |
| pTargetET | pEC891 carrying editing templates of *ilvA(805bp) and tdh(785bp),* initial acceptor receptor for CRISPR array assembly | This study |
| ptargetET-*tdh* | pTargetET carrying sgRNA-*tdh fragment* | This study |
| ptargetET-*tdh-ilvA* | pTargetET carrying sgRNA-*tdh* fragment and sgRNA-*ilvA* fragment | This study |
| p*thrA^433^BC*-*asd-kivD-ADH2* | *kivD* (Accession Number AJ746364) and *ADH2* of *Saccharomyces cerevisiae* coding sequence were under control of P_trc_ and BCD1. | This study |

Table S2 Primers used in this study with relevant characteristics.

| Primers | Sequences (5’-3’) | Function |
| --- | --- | --- |
| **For PS-Brick assembly** | | |
| UC709-F | TGCGTATTGGGCGCTCTTCCGCTTCCTCGCTCACTGACACGCTGCGCTCGGTCGTTCG | *Mly*I^T709A^ mutation |
| UC1179-R | GTCGTGTCTTACCGGGTTGGAATCAAGACG ATAGTTACCGGAT | *Mly*I^G1179T^ mutation |
| UC1179-F | ATCCGGTAACTATCGTCTTGATTCCAACCCGGTAAGACACGAC |  |
| UC1695-R | TGGTAAGCCCTCCCGTATCGTAGTTATCTACACGACGGGGAGCCAGGCAA CTATGGATG | *Mly*I^A1695G^ mutation |
| UC1746-R | AGCGTGGGTCTCGCGGTATC ATTGCAGCACTAGGGCCAGA TGGTAAGCCC TCCCGTATC | *Bmr*I^C1746T^ mutation |
| pUC19- *Mly*I^1177^-R | GTCGTGTCTTACCGGGTTGG AATCAAGACGATAGTTACCG GAT |  |
| UC1 | GCACAGATGCGTAAGGAGA | For sequencing verification of *Mly*I sites and BmrI site mutation, and the whole sequence of pO19 |
| UC2 | GCAGGAAAGAACATGTGAGCA |  |
| UC3 | AGGATCTTCACCTAGATCCT |  |
| UC4 | GTTCGATGTAACCCACTCGT |  |
| mC-F | GGGAATTCCATATGATGGTGAGCAAGGGCGAGGA (NdeI) | Amplification of truncated *mCherry* gene segment |
| mCB-R | ACATGCATGCACTGGGGAGGAGTCCTGGGTCACGGTCA (SphI/BmrI) |  |
| mCM-R | ACATGCATGCGAGTCGAGTAGTCCTGGGTCACGGTCA (SphI/*Mly*I) |  |
| FB-F | CCTCCCTGCAGGACGGCGAGT | FB amplification |
| FB-R | ACATGCATGCACTGGGTTACTTGTACAGCTCGTCCA (SphI/BmrI) |  |
| FM-F | TCCTCCCTGCAGGACGGCGAGT | FM amplification |
| FM-R | ACATGCATGCGAGTCTTACTTGTAC AGCTCGTCCA (SphI/*Mly*I) |  |
| UC5 | ACGGTGAAAACC TCTGACACA | For sequencing verification of pOB and pOM, and their ligation with FB and FM, respectively |
| UC6 | CGCAACGCAATTAATGTGAGT |  |
| **For metabolic pathway engineering** | | |
| AC^3211^-F | ACCACGATACTATGACTGAGTGTCAACGCCATGAGCGGCC TCA | For three *Mly*I sites mutation in pACYC184 vector |
| AC^727^-R | GAACGACCGAGCGTAGCGTG TCAGTGAGCG AGGAAG |  |
| AC^727^-F | CTTCCTCGCTCACTGACACGC TACGCTCGGT CGTTC |  |
| AC1143-R | AGTGGTGCTTTTGCATGTCTTTCCGGGTTGGAATCAAGAC GATAGTTACC GGATAAGGC |  |
| AC1 | GGCTTACTAT GTTGGCACTG | For sequencing verification of *Mly*I sites mutation and the whole sequence of pO19 |
| AC2 | CCTGTGGAAC ACCTACATCT |  |
| AC3 | GATCG TGCTCCTGTC GTTGA |  |
| TAB-F | CCCAAGCTTGAGTCAGGGATCTTCTGAACGCTCAATCTCT (*Hind*III/*Mly*I) | For fusion of *thrB*, *thrC* and truncated *thrA* sequence by overlap PCR. For Removing *Mly*I and *Hind*III sites in sequence which the color of target were marked to grey. |
| TAB-R | GGCATAAACTTTAACCATGTCAAACTCCTAACTTCCATGAGAGGGTACG |  |
| TBC-F | CGTACCCTCTCATGGAAGTTAGGAGTTTGACATGGTTAAAGTTTATGCC |  |
| TBC-R | GCTCACGTCCATCGCGTTGGATAACGTCGCCTGCGTCGCTTTGGGTGACCACTG |  |
| TC-F | GCAGGCGACGTT ATCCAACGCGATGGACGTGA GCCAGCCGAA CAACTGGC |  |
| TC-R | CCCTCGCGAGCATTTATTGAGAATTTCTCC (NruI) |  |
| TA-F | CCCAAGCTTGAGTCCTGGT CGACTGGTTACAACA (*Hind*III/*Mly*I) | For generating native and 20 different codon saturation mutagenesis in the 433th residue of ThrA |
| TA^phe^-R | AAACTGAGCAATGGCGACAATGT |  |
| TA^Leu^-R | CAGCTGAGCAATGGCGACAATGT |  |
| TA^Ile^-R | AATCTGAGCAATGGCGACAATGT |  |
| TA^Met^-R | CATCTGAGCAATGGCGACAATGT |  |
| TA^Val^-R | CACCTGAGCAATGGCGACAATGT |  |
| TA^Ser^-R | GCTCTGAGCAATGGCGACAATGT |  |
| TA^Pro^-R | CGGCTGAGCAATGGCGACAATGT |  |
| TA^Thr^-R | GGTCTGAGCAATGGCGACAATGT |  |
| TA^Ala^-R | CGCCTGAGCAATGGCGACAATGT |  |
| TA^Tyr^-R | ATACTGAGCAATGGCGACAATGT |  |
| TA^His^-R | ATGCTGAGCAATGGCGACAATGT |  |
| TA^Gln^-R | CTGCTGAGCAATGGCGACAATGT |  |
| TA^Asn^-R | GTTCTGAGCAATGGCGACAATGT |  |
| TA^Lys^-R | TTTCTGAGCAATGGCGACAATGT |  |
| TA^Asp^-R | ATCCTGAGCAATGGCGACAATGT |  |
| TA^Glu^-R | TTCCTGAGCAATGGCGACAATGT |  |
| TA^Cys^-R | GCACTGAGCAATGGCGACAATGT |  |
| TA^Trp^-R | CCACTGAGCAATGGCGACAATGT |  |
| TA^Arg^-R | TCTCTGAGCAATGGCGACAATGT |  |
| TA^Gly^-R | TCCCTGAGCAATGGCGACAATGT |  |
| aspC1-F | CCAGATCGAT TCTGACAACA | Overlapped *aspC* amplication with *Mly*I site mutated |
| aspC1-R | CCCGGAGTTTGTGCCGTGCG AGCAC |  |
| aspC2-F | GTGCTCGCACGGCACAAACT CCGGG |  |
| aspC2-R | CCCAAGCTTGAGTCCGTGCAAATTCAAAATATTGCA (*Hind*III/*Mly*I) |  |
| aspA-F | CAGCATATGATC TCGGGTATTC | *aspA* amplication |
| aspA-R | CCCAAGCTTGAGTC CTGCTCACAA GAAAAAAGGCA (*Hind*III/*Mly*I) |  |
| ppc1-F | CGACCT ACACCTTTGG TGT | Overlapped *ppc* amplication with *Mly*I sites mutated |
| ppc1-R | CGCATCTTTTGCTGAATCGG AATAGCCAAT CATC |  |
| ppc2-F | GATGATTGGCTATTCCGATT CAGCAAAAGA TGCG |  |
| ppc2-R | GGAATGGCGCGTAGTGATTC GACGCCG |  |
| ppc3-F | CGGCGTCGAATCACTACGCGCCATTCC |  |
| ppc3-R | TCCGTAGCTGAATAGATTCT GCAATCCACG GCAG |  |
| ppc4-F | CTGCCGTGGATTGCAGA ATCTATTCAG CTACGGA |  |
| ppc4-R | CCCAAGCTTGAGTCGAAAACGAGGGTGTTAGAACAG (*Hind*III/*Mly*I) |  |
| asd1-F | CTTTCTGCGTGCTAACAAAGCA | Overlapped *asd* amplication with *Hind*III site mutated |
| Asd1-R | CATCCGCTTTCACGGAGCTT TGGATAGATT TCG |  |
| Asd2-F | CGAAATCTATCCAAAGCTCCGTGA AAGCGGATG |  |
| Asd2-R | CCCAAGCTTGAGTCGCTCTATTTAACTCCCGGTAAATC (*Hind*III/*Mly*I) |  |
| pntA/B1-F | CCACTATCACGGCTGAATC | Overlapped *pntAB* amplication with *Mly*I sites mutated |
| pntA/B1-R | CGGCACAGAATCCATCGCCA TCACGGT |  |
| pntA/B2-F | ACCGTGATGGCGATGGATTC TGTGCCG |  |
| pntA/B2-R | GCCTTCATGGAATCAACCAT TTCACGGGT |  |
| pntA/B3-F | ACCCGTGAAATGGTTGAATCCATGAAGGC |  |
| pntA/B3-R | CAGCATGCGCTGAGTAACGG TGAAGCCACC GA |  |
| pntA/B4-F | TCGGTGGCTTCACCGTTACT CAGCGCATGC TG |  |
| pntA/B4-R | ACCAGCAATCGGACTTTTCG GATCATCCTG C |  |
| pntA/B5-F | GCAGGATGATCCGAAAA GTCCGATTGC TGGT |  |
| pntA/B5-R | CCCAAGCTTGAGTCTGGGTATGCT GCTTTCCGT (*Hind*III/*Mly*I) |  |
| rhtA-F | CCCAAGCTTGAGTCAAAGGATGCCTGGTTCATTACGT (*Hind*III/*Mly*I) | *rhtA* amplification |
| rhtA-R | CTAATAGTGGTAACAAGCGTGA |  |
| rhtB-F | CCCAAGCTTGAGTCTCATCATGACCTTAGAATGGTGGT (*Hind*III/*Mly*I) | *rhtB* amplification |
| rhtB-R | GCGTGGTTTACCGTCGTT |  |
| rhtC-F | CCCAAGCTTGAGTCAATGTATGTTGATGTTATTTCTCACCGT (*Hind*III/*Mly*I) | *rhtC* amplification |
| rhtC-R | CTTGCTCAAC GGATTGCTCT |  |
| yecC-F | CCCAAGCTTGAGTCCCAAAATGAGTGCCATTGAAGT (*Hind*III/*Mly*I) | *yecC* amplification |
| yecC-R | AGTTATGCTGATTTGTTAAGCAGT |  |
| T-F | CCCAAGCTTGAGTCCCAAACAATTCCGACGTCTAAGAAG (*Hind*III/*Mly*I) | Overlap PCR for generating pT-BCD1 fragment |
| TBCD-R | CTCCTTTTTAAGTGAACTTGGGCCCGGTCAGTGCGTCCTG CTGA |  |
| TBCD-F | TCAGCAGGACGCACTGACCGGGCCCAAGTTCACTTAAAAAGGAG |  |
| BCD-R | TAGAAAGTCT CCTGTGCATG A |  |
| Prop-F | ttgacaat taatcatccg gctcgt | Ptrc-BDC1-kivD-ADH2-Ter amplification |
| Prop-R | CCCAAGCTTGAGTCCAAAAAACCC CTCAAGACC (*Hind*III/*Mly*I) |  |
| **For CRISPR array** | | |
| TGB-F | AGGGAGAAAGGCGGACAGGTTTCCGGTAAGCGGCAGGGTC | For *Bci*VI site mutation in plasmid pTaregetF |
| TGB-R | GACCCTGCCGCTTACCGGAAACCTGTCCGC CTTTCTCCCT |  |
| N20-*tdcC*-F | TCCTAGGTATAATACTAGTCACCGGTCATGGACGCAAAGGTTTTAGAGCT AGAAATAGC | For generating pTargetF-*tdcC* plasmid |
| N20-*tdcC*-R | GCTATTTCTAGCTCTAAAACCTTTGCGTCCATGACCGGTGACTAGTATTA TACCTAGGA |  |
| N20-*ilvA*-F | TCCTAGGTATAATACTAGTCTTCATCAAAGTTCGCGCCGGTTTTAGAGCT AGAAATAGC | For generating pTargetF-*ilvA* plasmid |
| N20-*ilvA*-R | GCTATTTCTAGCTCTAAAACCGGCGCGAACTTTGATGAAGACTAGTATTA TACCTAGGA |  |
| N20-*tdh*-R | TCCTAGGTATAATACTAGTCTTTGGCGACGTTAACCGCAGTTTTAGAGCTAGAAATAGC | For generating pTargetF-*tdh* plasmid |
| N20-*tdh*-R | GCTATTTCTAGCTCTAAAACTGCGGTTAACGTCGCCAAAGACTAGTATTA TACCTAGGA |  |
| *tdc1*-F | CCCAAGCTTGTATCCCGCTTACCAGACAAAACAATCGTCCT (*Hind*III/*Bci*VI) | For amplification of upstream editing template of *tdcC* |
| *tdc1*-R | CCATGGCTTCAATCAGGTCCTGGTTTTCCCAGAACGTCA |  |
| tdc2-F | TGACGTTCTGGGAAAACCAGGA CCTGATTGAA GCCATGG | For amplification of downstream editing template of *tdcC* |
| tdc2-R | GTAGAGCATTTCCTGCATCCCCTTCAATGCACCTTCGTAG |  |
| ilv1-F | CTACGAAGGTGCATTGAAGGGGATGCAGGAAATGCTCTAC | For amplification of upstream editing template of *ilvA* |
| ilv1-R | GCGCTATCAGGCATTTTTCCTATTAACCCCCCAGTTTCGAT |  |
| ilv2-F | ATCGAAACTGGGGGGTTAATAGGAAA AATGCCTGAT AGCGC | For amplification of downstream editing template of *ilvA* |
| ilv2-R | AGTTGGAGAACAGGTACGGACGTAATCAGGTGTCGGTAGA |  |
| tdh^1^-F | TCTACCGACACCTGATTACGTCCGTACCTG TTCTCCAACT | For amplification of upstream editing template of *tdh* |
| tdh^1^-R | GAATACCAGCCCTTGTTCGTCTCACATCCTCAGGCGATAA |  |
| tdh^2^-F | TTATCGCCTGAGGATGTGAGACG AACAAGGGCT GGTATTC | For amplification of downstream editing template of *tdh* |
| tdh^2^-R | CGCGGATCCCAGAATTATCCGTTGAACCATCGT (*Bam*HI) |  |
| sgRNA-F | CCCAAGCTTGTATCCCGCTTACCTTGACAGCTAGCTCAGT (*Hind*III/*Bci*VI) | Amplification of pJ23119 promotor and gRNA scaffold sequence for CRISPR array |
| sgRNA-R | TGCAGGTCGA CTCTAGAGA |  |
| TG-F | GAACTCGAGT AGGGATAACAG | Forward primer for verification of ligation accuracy incorporating with WY3355 |
| ilvA-I-F | ACGATGCG GTAGAAGCGA TTCT | Chromosome *ilvA* identification |
| ilvA-I-R | GAGAATCTGGCAGTAGTGCTGAT |  |
| *tdh*-I-F | ATATTATCAC CGGTACGCTT GGT | Chromosome *tdh* identification |
| *tdh*-I-R | GCCTGATGCAACAAACGAACGT |  |
| **For RT-qPCR** | | |
| RT*aspA*-F | TGTCAGTCCACTAACGACGC |  |
| RT*aspA*-R | AGCCTTCACGCAGTTGGTTA |  |
| RT*aspC*-F | CTGCAGGTCTGGAAGTTCGT |  |
| RT*aspC*-R | CGTAGGGTCGATACCGGTTG |  |
| RT*ppC*-F | CGCTGCAAAAAGTGGTCGAA |  |
| RT*ppC*-R | TTGTCTACCAGGCGTTGGTC |  |
| RT*asd*-F | GTTGATTGGGTGTCCGTTGC |  |
| RT*asd*-R | CCGCTACGGGTTAAGGTTGT |  |
| RT*pntA*-F | TTTTTGGCTGGATGGCAAGC |  |
| RT*pntA*-R | ATCCCTGAAATCGCGTTGGT |  |
| RT*pntB*-F | TTCAGTCTGGCCGGTCTTTC |  |
| RT*pntB*-R | GGATACCAATTGCCCCACCA |  |
| RT*rhtA*-F | GGCACTGGTCGGTTATTCCA |  |
| RT*rhtA*-R | AGCGTACCAAATGTCCGTGT |  |
| RT*rhtB*-F | CAGTATATCGTGCTCGGCGT |  |
| RT*rhtB*-R | ATTCAGCGCCTTCATCTGCT |  |
| RT*rhtC*-F | ATGCTACGTGGTGCACTGAA |  |
| RT*rhtC*-R | TGCCAACGTTATCACCGACA |  |
| RT*yecC*-F | TGAGGTAAAGCCTGGCGAAG |  |
| RT*yecC*-R | TCTGGAAGACAAACCCGACG |  |
| RT*GapA*-F | GATGGCCCGTCTCACAAAGA |  |
| RT*GapA*-R | CAGACGAACGGTCAGGTCAA |  |

1. Angeles TS, Smanik PA, Borders CL, Jr., Viola RE. Aspartokinase-homoserine dehydrogenase I from Escherichia coli: pH and chemical modification studies of the kinase activity. Biochemistry. 1989;28:8771-7.

2. Mutalik VK, Guimaraes JC, Cambray G, Lam C, Christoffersen MJ, Mai QA, Tran AB, Paull M, Keasling JD, Arkin AP *et al*. Precise and reliable gene expression via standard transcription and translation initiation elements. Nat Methods. 2013;10:354-+.

3. Jiang Y, Chen B, Duan CL, Sun BB, Yang JJ, Yang S. Multigene Editing in the Escherichia coli Genome via the CRISPR-Cas9 System. Appl Environ Microb. 2015;81:2506-14.

4. Jiang Y, Chen B, Duan C, Sun B, Yang J, Yang S. Multigene editing in the Escherichia coli genome via the CRISPR-Cas9 system. Applied and environmental microbiology. 2015;81:2506-14.
